# Bis-Tridendate Ir(III) Polymer-Metallocomplexes: Hybrid, Main-Chain Polymer Phosphors for Orange–Red Light Emission

**DOI:** 10.3390/polym12122976

**Published:** 2020-12-13

**Authors:** Konstantinos Andrikopoulos, Charalampos Anastasopoulos, Joannis K. Kallitsis, Aikaterini K. Andreopoulou

**Affiliations:** 1 Department of Chemistry, University of Patras, University Campus Rio-Patras, GR26504 Patras, Greece; k.andrikopoulos@upnet.gr (K.A.); xanastasop@upatras.gr (C.A.); j.kallitsis@upatras.gr (J.K.K.); 2 Foundation for Research and Technology, Hellas/Institute of Chemical Engineering Sciences (FORTH/ICE-HT), Platani Str., GR26504 Patras, Greece

**Keywords:** polymeric metallocomplexes, iridium tridentate complexes, organic light-emitting diodes, solution-processable iridium polymers

## Abstract

In this work, hybrid polymeric bis-tridentate iridium(III) complexes bearing derivatives of terpyridine (tpy) and 2,6-di(phenyl) pyridine as ligands were successfully synthesized and evaluated as red-light emitters. At first, the synthesis of small molecular bis-tridendate Ir(III) complexes bearing alkoxy-, methyl-, or hydroxy-functionalized terpyridines and a dihydroxyphenyl-pyridine moiety was accomplished. Molecular complexes bearing two polymerizable end-hydroxyl groups and methyl- or alkoxy-decorated terpyridines were copolymerized with difluorodiphenyl-sulphone under high temperature polyetherification conditions. Alternatively, the post-polymerization complexation of the terpyridine-iridium(III) monocomplexes onto the biphenyl-pyridine main chain homopolymer was explored. Both cases afforded solution-processable metallocomplex-polymers possessing the advantages of phosphorescent emitters in addition to high molecular weights and excellent film-forming ability via solution casting. The structural, optical, and electrochemical properties of the monomeric and polymeric heteroleptic iridium complexes were thoroughly investigated. The polymeric metallocomplexes were found to emit in the orange–red region (550–600 nm) with appropriate HOMO and LUMO levels to be used in conjunction with blue-emitting hosts. By varying the metal loading on the polymeric backbone, the emitter’s specific emission maxima could be successfully tuned.

## 1. Introduction

Complexes involving iridium have shown great promise as organic light-emitting diode (OLED) emitters due to their triplet harvesting properties, increasing the theoretical limit of the external quantum efficiency (EQE) of the final device to 100% through the utilization of triplet excitons [1,2,3]. Furthermore, iridium(III) complexes have been intensively studied as emitters in light-emitting electrochemical cells (LEECs) [4,5], as imaging or sensing agents [6], in photo-catalysis [7], and even as light absorbers in dye-sensitized solar cells [8]. Regardless of the end-use application of iridium complexes, the complexation modes mainly involve tris- or bis-cyclometalated compounds depending on the choice of bidentate or tridentate ligands, respectively [9].

Of these two families, tris-cyclometalated iridium(III) complexes have been more prominently featured in the literature, as they offer facile modification routes and color tunability [10,11,12,13]. Such tris-bidentate iridium polyimine complexes, like bis[2-(4,6-difluorophenyl)pyridinato-C2,N](picolinato)iridium(III) (FIrpic), tris[2-phenylpyridinato-C2,N]iridium(III)(Ir(ppy)_3_), bis(4-phenyl-thieno[3,2-c]pyridinato-C2,N)(acetylacetonato)iridium(III)(PO-01), and bis(2-methyldibenzo[f,h]quinoxaline)(acetylacetonate)iridium(III) (Ir(MDQ)_2_(acac)), have been extensively used in light-emitting devices with high EQEs [14,15]. However, the existence of regioisomerism in these tris-bidentate iridium complexes requires tedious separations of the more stable isomers prior to their incorporation in the OLED’s active layer.

On the other hand, bis-tridentate iridium complexes have only seldom been incorporated in OLED devices due to poor reaction yields [16,17,18,19] and average device performances. However, they have been investigated as luminescent probes [20], photoactive assemblies [21,22], and as candidates for photodynamic therapy [23]. Regarding the use of bis-tridentate iridium complexes as light emitters, a series of bis-tridentate Ir(III) complexes with a (N^N^N)-(N^N^N) mode consisting of a series of terpyridine derivatives modified at the fourth position of the central pyridine unit [16] have been reported. The preparation of those red-emitting complexes (600 nm) involved the stepwise introduction of the two ligands in consecutive steps, with the second ligand requiring high reaction temperatures of 180 °C and above. Furthermore, and in order to raise the device efficiencies of bis-tridentate Ir(III) complexes used as dopants in the emitting layers (EMLs) of OLEDs, carbene-based ligands have been of special interest lately because of their increased luminous efficiency [24,25,26,27]. In general, bis-tridentate complexes require harsh reaction conditions to incorporate the second ligand, as the Ir(III) center displays a very stable coordination sphere. Additionally, the yields of the final complexes usually remain low because the scrambling of the ligands is often observed, and this leads to product mixtures that require chromatographic purification [19].

Other complexation modes have been also explored in an effort to tune the emission color and stability of the iridium complexes, mainly involving polyimine chelates with one or more pyridyl rings and complementary phenyl rings [19,28,29,30,31]. In particular, carbene complexes are considered to be promising alternatives to traditional tridentate chelates because they display increased emission efficiencies, with EQEs up to even 31%, due to the increased strength of the Ir-C_carbene_ bond that destabilizes the d–d transitions of the metal center [31,32].

As explained above, solution-processable small molecular Ir(III) complexes have been widely employed as “guest” molecules in combination with “host” polymers according to their energy levels and color emissions. On the contrary, only a handful solution-processable Ir(III)-bearing polymers have been identified as potential candidates, whether as standalone or blend components, for the EMLs of OLEDs [33]. In general, polymer metallocomplexes are an attractive solution, as they uniquely combine the advantages of small-molecular complexes with polymers’ superior film-forming properties and excellent thermal and chemical stability. Polymers containing iridium complexes, either along the main chain or as pendant groups, have been developed, mainly with the involvement of tris-bidentate complexes, affording polymeric metallocomplexes with a variety of emission colors [34,35,36,37,38,39]. However, bis-tridentate polymeric metallocomplexes are scarcer in the literature. Aamer et al. [40] investigated the synthesis of side chain-complexated Ir(III) block copolymers bearing terpyridine ligands. Their synthetic strategy involved either the synthesis of a precursor block copolymer, P(S-b-AA), and the subsequent modification of the AA units with NH_2_ functionalized terpyridines or the synthesis of the asymmetric complex and the subsequent modification of the block copolymer with an amide bond-forming reaction. Overall, while main chain tris-bidentate Ir(III) polymeric metallocomplexes have been the subject of research by several groups [36,41,42,43,44], their bis-tridentate counterparts have not been explored yet, to the best of our knowledge.

Inspired by this gap, in this work, we developed bis-tridentate Ir(III) complexes as small-molecular heteroleptic complexes and, most importantly, as polymer metallocomplexes of various complexation degrees. These complexes comprise phenyl terpyridine-Ir(III)-diphenyl pyridine central moieties that correspond to an Ir(N^N^N)(C^N^C) complexation mode in order to afford orange–red-emitting complexes. In order to facilitate the solubility and processability of the mono-molecular complexes and, most importantly, of their respective polymer metallocomplexes, as well as to ensure the blocking of interchain energy transfer [45], three terpyridine-based ligands were employed, modified with a hydroxyl, a dodecyloxy, or a methyl group at the 4′ position of the phenyl terpyridine ligand. The preparation of the polymer metallocomplexes followed two routes: (i) the direct polymerization of a dihydroxy-phenyl monomeric Ir(III) complex that was polymerized under high temperature polyetherification conditions and (ii) the post polymerization complexation of the Ir(III) monocomplexes of either dodecyloxyphenyl terpyridine or methylphenyl terpyridine with the free diphenylpyridines along the backbone of the diphenylpyridine–diphenylsulfone homopolymer. This latter case led to copolymers combining pure organic and organic–metallocomplex repeating units, allowing for different loads of iridium(III) and ensuring processability, high molecular weights, and film formation. The excellent solubility of most of the herein-synthesized small molecular complexes and polymeric metallocomplex materials allowed for their detailed structural, optoelectronic, and electrochemical characterization. All tridentate complexes showed red–orange emissions, with emission maxima in the range of 565–610 nm depending on the detailed chemical structure of the complexes, the complexation degree of the metallopolymers, and the processing conditions.

## 2. Materials and Methods

### 2.1. Materials

4(Phenyl-4-hydroxy) terpyridine (**HOtpy**) [46], 4 (phenyl-4-dodecyloxy) terpyridine (**C_12_Otpy**) [46], 2,6-Bis(4-hydroxyphenyl)pyridine (**HOpy**) [47], and the homopolymer (**pySO_2_**) [48] were prepared according to literature procedures. Bis(4-fluorophenyl)sulfone (**diFSO_2_**) was purchased from Chemos GmbH (Altdorf, Germany). Iridium(III) chloride hydrate was purchased from Fluorochem (Hadfield Derbyshire, UK). All other solvents and reagents were purchased from Aldrich ((Sigma-Aldrich Chemie GmbH, Taufkirchen, Germany)), Acros (Morris Plains, NJ, USA), Alfa Aesar (Thermo Fisher (Kandel) GmbH Postfach, Karlsruhe, Germany), or Merck (Taufkirchen, Germany) and used without further purification, unless otherwise stated.

### 2.2. Instrumentation

^1^H-NMR and proton-decoupled ^13^C-NMR spectra [^13^C-{^1^H}] were obtained at 600 and 150 MHz, respectively, on a Bruker AvanceIII HD spectrometer (Bruker BioSpin GmbH, Magnet Division, Karlsruhe, Germany) at 25 °C using deuterated CDCl_3_ or d_6_-DMSO. Chemical shifts (δ) are reported in units, parts per million (ppm) downfield from TMS. The abbreviations used in the description of the NMR data are as follows: br, broad; s, singlet; d, doublet; t, triplet; and m, multiplet.

Matrix-assisted laser desorption/ionization time-of-flight mass spectrometry (MALDI-TOF MS) and laser desorption/ionization time-of-flight mass spectrometry (LDI-TOF MS) were performed using an Autoflex Speed MALDI-TOF/TOF mass spectrometer from Bruker Daltonics GmbH (Bremen, Germany) in the positive reflection mode using either a-cyano-4-hydroxycinnamic acid (HCCA) as the matrix or the matrix-free approach.

Size-exclusion chromatography (SEC) measurements were carried out using a Polymer Lab chromatographer (Agilent Technologies, Santa Clara, CA, USA) equipped with two PLgel 5 μm mixed columns and a UV detector using CHCl_3_ as the eluent at a flow rate of 1 mL min^−1^ at 25 °C; and calibrated versus polystyrene standards.

Attenuated total reflectance Fourier transform infrared spectra (ATR-FTIR) were recorded on a “Bruker Optics’ Alpha-P Diamond ATR Spectrometer of Bruker Optics GmbH” (Ettlingen, Germany).

The UV–vis spectra were recorded using a Hitachi-Science and Technology-U-1800 UV–vis Spectrophotometer (Hitachi High-Technologies Europe GmbH, Mannheim, Germany). Photoluminescence spectra were recorded using a Perkin Elmer LS45 Fluorescence Spectrometer (Waltham, MA, USA).

Cyclic voltammetry was performed using an Autolab Potentiostat (Utrecht, The Netherlands) in HPLC-grade acetonitrile (CH_3_CN) using tetrabutylammonium hexafluorophosphate (TBAPF_6_) as the supporting electrolyte in 0.1 M concentration with a scan rate of 100 mV/s. The working electrode was a fluorine-doped indium-tin oxide glass substrate, and the counter electrode was a Pt wire. The reference electrode was an Ag/AgCl electrode, and the electrochemical pair of ferrocene/ferrocenium ion was used as a standard. The compound for each measurement was drop-cast onto the working electrode and dried overnight at 80 °C. Before carrying out the measurements, the cell was purged with pure argon for 20 min to remove diluted gasses. The LUMO energy levels were calculated from the first reduction onset potential using the equation:

ELUMO = −e (E_red,onset_ − E_1/2Ferrocene_) − 5.2 [eV] (1)

where E_red,onset_ is the onset determined for the reduction peak of each molecule in cyclic voltammetry (V) versus Ag/Ag^+^.

E_1/2Ferrocene_ = (E_red_ + E_ox_)/2 vs. Ag/AgCl (2)

The HOMO energy levels were determined from the equation:

E_HOMO_ = −e (E_ox,onset_ − E_1/2Ferrocene_) − 5.2 [eV] (3)

where E_ox,onset_ is the onset determined for the oxidation peak of each molecule in cyclic voltammetry (V) versus Ag/Ag^+^.

### 2.3. Synthesis of Compounds

#### 2.3.1. Monocomplexes R-tpy-IrCl_3_

**HOtpy-IrCl_3_:** In a round-bottom flask after it was Ar-purged, iridium chloride hydrate (77 mg; 0.26 mmol) and 4-(phenyl-4-hydroxy) terpyridine (HOtpy) (84 mg; 0.26 mmol) were dissolved in a mixture of EtOH and THF. The reaction mixture was stirred at reflux for 5 h. Then, the mixture was allowed to cool to room temperature before the produced solid was filtered off and washed with ethanol, toluene, and water. The final HOtpy-IrCl_3_ was dried for 10 h at 50 °C under vacuum, thus affording 150 mg (75% yield). ^1^H NMR (600 MHz, DMSO-d_6_, *δ* ppm): 9.96 (s, 1H), 8,81 (d, J = 3.87 Hz, 2H), 8.78 (d, J = 7.73 Hz, 2H), 8.71 (s, 2H), 8.16 (t, 7.16 Hz, 2H), 7.85 (d, J = 8.36 Hz, 2H), 7.63 (t, J = 5.33 Hz, 2H), 6.98 (d, J = 8.39 Hz, 2H). ^13^C-{^1^H} NMR (150 MHz, DMSO-d_6_, *δ* ppm): 162.77, 159.82, 157.51, 153.42, 151.49, 140.61, 130.61, 130.33, 128.91, 128.65, 127.22, 126.09, 125.63, 120.15, 116.68, 116.48. MALDI-TOF MS calculated for C_21_H_15_Cl_3_IrN_3_O m/z: 622.99, found: 552.79 [M-H-2Cl], 587.78 [M-H-Cl], 706.93 [M+K-H], 841.04 [M+HO-tpy ligand].

**C_12_Otpy-IrCl_3_:** The same procedure as HOtpy-IrCl_3_ was followed, starting from 4(phenyl-4-dodecyloxy)terpyridine(C_12_Otpy) to afford 120 mg of C_12_Otpy-IrCl_3_ (60% yield). ^1^H NMR (600 MHz, DMSO-d_6_, *δ* ppm): 8.88–8.81 (m, 4H), 8.75 (s, 2H), 8.22 (t, J = 6.59 Hz, 2H), 7.94 (d, J = 7.87 Hz, 2H), 7.69 (t, J = 5.17 Hz, 2H), 7.14 (d, J = 8.08 Hz, 2H), 4.08 (t, J = 5.91 Hz, 2H), 1.77 (t, J = 6.93 Hz, 2H), 1.46 (t, J = 6.55 Hz, 2H), 1.37–1.27 (m, 16H), 0.86 (t, J = 5.59 Hz, 3H). ^13^C-{^1^H} NMR (150 MHz, DMSO-d_6_, *δ* ppm): 161.68, 160.37, 159.76, 158.69, 157.59, 156.04, 155.50, 154.57, 153.39, 151.12, 149.77, 143.06, 142.65, 140.66, 137.93, 131.15, 130.79, 130.54, 130.26, 129.99, 129.80, 128.68, 128.63, 127.93, 127.58, 127.37, 125.74, 124.95, 122.90, 122.88, 121.38, 120.36, 117.68, 115.82, 115.75, 115.69, 115.61, 115.56, 68.48, 68.30, 68.10, 34.81, 31.72, 29.47, 29.44, 29.42, 29.29, 29.20, 29.14, 29.04, 26.10, 25.92, 22.53, 14.39. MALDI-TOF MS: calculated for C_33_H_39_Cl_3_IrN_3_O m/z: 791.18, found: 721.17 [M-2Cl^-^], 756.15 [M-Cl], 1177.63 [M+C_12_H_25_Otpy ligand].

**CH_3_tpy-IrCl_3_:** The same procedure as HOtpy-IrCl_3_ was followed, starting from 4′-(4-methylphenyl)-2,2′:6′,2″-terpyridine (CH_3_tpy) to afford 284 mg of CH_3_tpy-IrCl_3_ (70% yield). ^1^H NMR (600 MHz, DMSO-d_6_, *δ* ppm): 9.2 (dd, J = 5.53, 0.92 Hz, 2H), 9.06 (s, 1H), 8.90–8.85 (m, 2H), 8.77 (s, 1H), 8.29–8.26 (m, 2H), 8.12 (d, J = 8.01 Hz, 2H), 7.95 (t, J = 6.48 Hz, 2H), 7.90 (d, J = 7.44 Hz, 2H), 7.72 (s, 1H), 7.48 (d, J = 7.93 Hz, 2H), 7.41 (d, J = 7.39, 2H), 2.46 (s, 3H), 2.39 (s, 3H). ^13^C-{^1^H} NMR (150 MHz, DMSO-d_6_, *δ* ppm): 162.82, 159.68, 157.75, 153.76, 153.72, 153.42, 152.81, 152.77, 151.42, 150.61, 147.88, 147.86, 141.54, 140.98, 140.95, 140.67, 140.27, 134.17, 132.61, 130.47, 130.28, 128.72, 128.52, 127.45, 126.10, 125.76, 122.98, 120.92, 119.61, 21.32. MALDI-TOF MS: calculated for C_22_H_17_ Cl_3_IrN_3_ m/z: 621.01, found: 550.88 [M-3Cl], 585.88 [M-Cl], 837.19 [M+CH_3_tpy ligand].

#### 2.3.2. Dicomplexes R-tpy-Ir-HOpy

**HOtpy-Ir-HOpy:** HOtpy-IrCl_3_ (100 mg; 0.1604 mmol) and 2,6-bis(4-hydroxyphenyl)-pyridine (HOpy) (46.5 mg; 0.1764 mmol) were added in a round-bottomed flask that was previously degassed and filled with Ar. The solids were dissolved in ethylene glycol and stirred at 200 °C for 2 h in the dark. Then, the reaction mixture was poured into an NH_4_PF_6_(aq) solution to precipitate the target complex. The precipitated solid was filtered off under vacuum and washed with water, toluene, chloroform, and diethyl ether. Then it was dried for 10 h at 50 °C under vacuum. The crude solid was recrystallized using an equimolar mixture of toluene-diethyl ether and then recrystallized again from an equimolar mixture of toluene-hexane, thus affording 44 mg of the target complex **HOtpy-Ir-HOpy** (35% yield). ^1^H NMR (600 MHz, DMSO-d_6_, *δ* ppm): 10.18 (s, 1H), 9.69 (s, 2H), 9.22 (d, J = 4.94 Hz, 2H), 9.01 (s, 2H), 8.89 (d, J = 7.88 Hz, 2H), 8.28 (q, J = 7.31 Hz, 2H), 8.10 (d, J = 8.6 Hz, 2H), 8.03 (d, J = 8.64 Hz, 2H), 7.95 (t, J = 6.16 Hz, 1H), 7.83–7.79 (two d, J = 8.17 Hz and 7.83 Hz, 2H), 7.72–7.67 (m, 4H), 7.04 (d, J = 8.63 Hz, 2H), 6.88 (d, J = 8.66 Hz, 2H). ^13^C-{^1^H} NMR (150 MHz, DMSO-d_6_, *δ* ppm): 167.67, 165.35, 160.90, 160.36, 159.81, 158.94, 158.79, 157.50, 157.24, 155.87, 153.42, 152.16, 151.49, 150.32, 146.42, 140.58, 140.36, 138.27, 137.39, 137.81, 130.61, 130.32, 129.82, 128.64, 128.38, 128.20, 127.90, 126.85, 126.04, 125.67, 125.60, 120.22, 120.11, 119.77, 116.93, 116.66, 116.48, 115.92, 114.02, 111.37. MALDI-TOF MS: calculated for C_38_H_26_IrN_4_O_3_ m/z: 779.16, found: 779.93 [M+H], 778.88 [M], 777.92 [M-H], 776.91 [M-3H].

**C_12_Otpy-Ir-HOpy:** The same procedure as above for HOtpy-Ir-HOpy was followed to afford 160 mg of C_12_Otpy-Ir-HOpy (40% yield). ^1^H NMR (600 MHz, DMSO-d_6_, *δ* ppm): 9.70 (s, 2H), 9.40 (s, 2H), 9.08 (d, J = 8.17 Hz, 2H), 9.04 (s, 2H), 8.38 (d, J = 8.57 Hz, 2H), 8.11 (t, J = 7.70 Hz, 2H), 7.94 (d, J = 6.70 Hz, 2H), 7.84 (d, J = 8.07Hz, 2H), 7.72 (m, 4H), 7.43 (t, J = 6.67 Hz, 1H), 7.28 (d, J = 8.46 Hz, 2H), 6.30 (d, J = 8.50 Hz, 2H), 4.16 (t, J = 6.29 Hz, 2H), 1.80 (t, J = 7.01 Hz, 2H), 1.49 (t, J = 5.35 Hz, 2H), 1.38–1.27 (m, 16H), 0.86 (t, J = 4.38 Hz, 3H). ^13^C-{^1^H} NMR (150 MHz, DMSO-d_6_, *δ* ppm): 167.73, 162.77, 161.59, 160.75, 159.43, 158.24, 157.15, 155.49, 154.36, 153.22, 152.54, 142.70, 141.31, 139.69, 138.45, 138.20, 130.12, 129.84, 129.60, 129.46, 129.03, 128.95, 127.15, 125.70, 123.09, 122.01, 121.09, 121.03, 117.23, 115.79, 115.46, 115.39, 115.34, 68.56, 68.39, 31.62, 29.33, 29.05, 28.85, 25.67, 22.36, 13.34. MALDI-TOF MS: calculated for C_50_H_50_IrN_4_O_3_ m/z: 947.35, found: 947.31 [M], 945.30 [M-2H].

**CH_3_tpy-Ir-HOpy:** The same procedure as above for HOtpy-Ir-HOpy was used to afford 150 mg of CH_3_tpy-Ir-HOpy (35% yield). ^1^H NMR (600 MHz, DMSO-d_6_, *δ* ppm): 9.60 (br s, 2H), 9.23 (d, J = 4.24 Hz, 2H), 9.09 (s, 2H), 9.03 (s, 2H), 8.96 (d, J = 7.40 Hz, 2H), 8.87 (d, J = 6.6 Hz, 2H), 8.41 (t, J = 7.06, 2H), 8.26 (t, J = 6.66 Hz, 2H), 8.1 (two d, J = 7.01 Hz and J = 7.54 Hz, 2H), 8.01 (d, J = 8.26 Hz, 2H), 7.78 (t, J = 7.22 Hz, 1H), 7.48 (m, 4H), 2.68 (s, 3H). ^13^C-{^1^H} NMR (150 MHz, DMSO-d_6_, *δ* ppm): 162.81, 159.04, 158.78, 157.12, 155.78, 152.68, 152.45, 141.78, 138.60, 130.47, 130.41, 130.34, 129.98, 129.91, 128.61, 128.51, 128.06, 125.72, 121.48, 119.76, 117.19, 115.95, 117.19, 115.95, 34.86. MALDI-TOF MS: calculated for C_39_H_28_IrN_4_O_2_ m/z:777.18, found: 776.93 [M], 775.93 [M-H], 774.93 [M-2H].

#### 2.3.3. Copolymer Metallocomplexes CPOL-Rtpy-Ir

**a-CPOL-Rtpy-Irx:** In a round bottom flask after it was purged with Ar, Rtpy-IrCl_3_ and the homopolymer pySO_2_ were dissolved in a mixture of NMP and ethylene glycol at reflux. The amount of each reactant was based on the desired metal loading of the final product. The reaction mixture was left in the dark for 2 h. Then, the crude mixture was poured into ethanol and NH_4_PF_6_(aq) and left stirring overnight. Then, the polymeric complex was filtered off and washed with ethanol, acetonitrile, and water. The final copolymeric metallocomplexes were obtained in yields ranging from 50% to 80%.

**b-CPOL-Rtpy-Irx:** Rtpy-Ir-HOpy and 2,6-bis(4-hydroxyphenyl)pyridine (HOpy), in amounts based on the final desired metal loading of the polymeric metallocomplex, were taken in a round bottom flask that was first degassed and purged with Ar. Then, K_2_CO_3_ and bis(4-fluoro)sulfone(diFSO_2_) were also added. The reactants were dissolved in a mixture of DMA and toluene. The flask was then fitted with a Dean–Stark apparatus and left to stir at 140 °C. After 4 h, the azeotropic mixture of toluene/water was removed from the reaction mixture, and this was left to further polymerize for another 4 h. After the completion of the polymerization reaction, the crude polymerization mixture was cooled to room temperature, poured into a saturated ethanol/water (10/1) solution of NH_4_PF_6_, and left to stir overnight. The thus obtained solid was filtered off and washed with water, ethanol, acetonitrile, and hexane. The final polymeric metallocomplex was dried under vacuum at 50 °C overnight to give a red powder in yields close to 40%.

For **a-CPOL-CH_3_tpy-Irx**: ^1^H NMR (600 MHz, DMSO-d_6_, *δ* ppm): 9.21 (m, 2H(x)), 9.07 (s, 2H(x)), 8.90 (d, J = 6.39 Hz, 2H(x)), 8.32–8.19 (br, 8H(x) and 4H(1-x)), 7.90 (br, 5H(x) and 8H(1-x)), 7.49 (m, 2H(x)), 7.29 (d, J = 8.47, 2H(x)), 7.1 (br, 8H(x) and 8H(1-x)), 2.68 (s, 3H(x)).

For **a-CPOL-C_12_Otpy-Irx**: ^1^H NMR (600 MHz, DMSO-d_6_, *δ* ppm): 9.20 (m, 2H(x)), 9.04 (s, 2H(x)), 8.89 (d, J = 7.68 Hz, 2H(x), 8.32–8.12 (br, 8H(x) and 4H(1-x)), 7.98–7.90 (br, 5H(x) and 8H(1-x)), 7.29–7.15 (br, 12H(x) and 8H(1-x)), 4.10 (br, 2H(x)), 2.16 (br, 2H(x)), 1.88 (br, 2H(x)), 1.43 (br, 2H(x)), 1.34-1.23 (br, 14H(x)), 0.83 (br, 3H(x)).

## 3. Results

### 3.1. Synthesis of Compounds

**Scheme 1:**
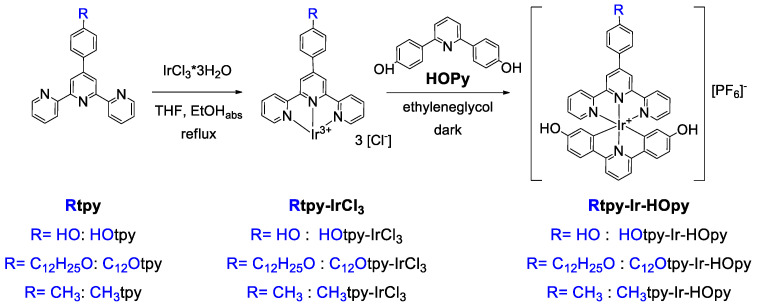
Synthesis of the functional-terpyridine-Ir(III)-diphenylpyridine (**Rtpy-Ir-HOpy**) heteroleptic monomer complexes.

Scheme 1 shows the two-step synthetic route of the terpyridine-Ir(III)-biphenylpyridine heteroleptic complexes carrying two hydroxylphenyl groups onto the biphenylpyridine moiety. The organic [49,50] ligands were prepared following literature procedures, also based on our previous know-how regarding polymeric structures involving terpyridines as side chain-tethering units for Ru(III) complexes [46,51]. According to the procedure of Scheme 1, iridium was first reacted with the terpyridine ligand with a hydroxylphenyl-(**HOtpy**), a dodecyloxyphenyl-(**C_12_Otpy**), or a methylphenyl-(**CH_3_tpy**) moiety in a mixture of ethanol and tetrahydrofuran at reflux to obtain the crude substituted monocomplexes [16]. The dodecyloxy- and methyl-functionalized terpyridines, **C_12_Otpy** [46] and **CH_3_tpy**, were employed to increase the solubility of the monomeric heteroleptic complex and, more importantly, to ensure the solubility of the polymeric metallocomplexes, as is shown below. Without further purification [16,18], the monocomplexes reacted with the second ligand, namely the 2,6-dihydroxylphenyl-pyridine (**HOpy**) [47], in refluxing ethyleneglycol to obtain the bis-tridentate complexes **HOtpy-Ir-HOpy**, **C_12_Otpy-Ir-HOpy**, and **CH_3_tpy-Ir-HOpy**. All complexation reactions were performed in the dark to avoid side reactions [16,18]. The purification of the monomeric dicomplexes was achieved with consecutive recrystallizations from mixtures of methanol-diethylether and hexane-toluene. All small molecular complexes showed solubility in a variety of organic solvents, although, as was anticipated, the long alkoxy chain of the **C_12_Otpy-Ir-HOpy** complex led to an enhanced solubility compared to the **HOtpy-Ir-HOpy** and the **CH_3_tpy-Ir-HOpy** complexes under the same solvent, concentration, and temperature conditions.

**Scheme 2:**
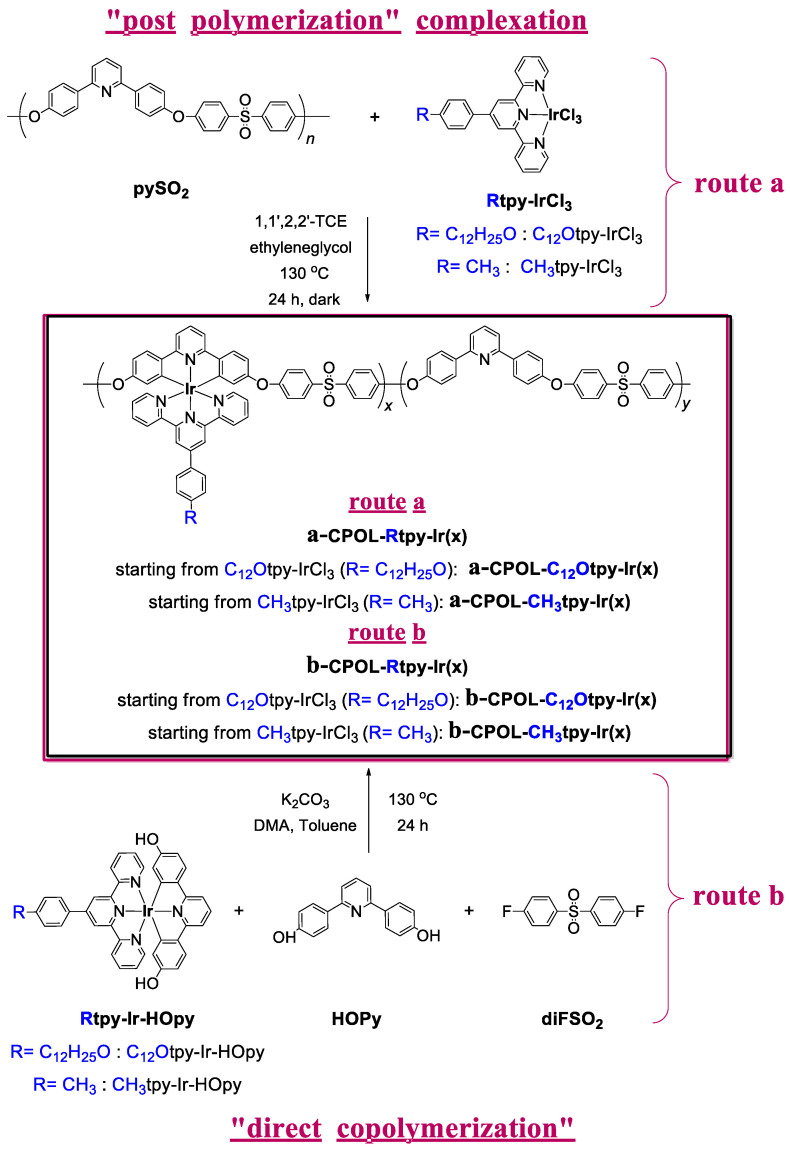
Synthesis of copolymer metallocomplexes: route a via the “post-polymerization” complexation of the homopolymer-ligand **pySO_2_** with the **C_12_Otpy-IrCl_3_** or **CH_3_tpy-IrCl_3_** monocomplexes, thus affording copolymers **a-CPOL-Rtpy-Ir(x)**, where **x** indicates the complexation degree; route b via the “direct copolymerization” of complexes **C_12_Otpy-Ir-HOpy** or **CH_3_tpy-Ir-HOpy** with the uncomplexed **HOpy** diol and the difluorophenyl-sulfone (**diFSO_2_**), thus affording copolymer metallocomplexes **b-CPOL-Rtpy-Ir(x)**.

For the development of polymeric metallocomplexes, two distinct strategies were used; direct co-polymerization of the dihydroxyl-functional monocomplexes or post-polymerization complexation (Scheme 2). For simplicity reasons, in the polymer metallocomplexes’ chemical structure of Scheme 2, the counterions (PF_6_^−^) have been omitted.

At first, as shown in Scheme 2—route a, a “post-polymerization” complexation approach was selected. In this, the diphenyl pyridine moieties of the homopolymer (**pySO_2_**) [48], prepared from 2,6-dihydroxyphenylpyridine (**HOpy**) [47] and difluorophenylsulfone (**diFSO_2_**), were complexated with the terpyridine-Ir monocomplexes with dodecyloxy or methyl side groups, **C_12_Otpy-IrCl_3_** and **CH_3_tpy-IrCl_3_**, respectively. Various percentages of complexation were investigated to transfer the photophysical properties of the monomeric complexes to the copolymeric metallocomplexes. These copolymers are denoted as **a-CPOL-C_12_Otpy-Ir(x)** or **a-CPOL-CH_3_tpy-Ir(x)**, where **x** stands for the complexation degree equivalent to the Ir content in the polymer. Efforts to incorporate the hydroxyl functional iridium monocomplex **HOtpy-IrCl_3_** were mostly accompanied with negligible solubility and insufficiently purified materials. Therefore, for all metallocomplex copolymers, only the dodecyloxy- and methyl-phenyl terpyridine ligands were studied.

In order to evaluate the effect of the synthetic methodology on the yield, purity, and properties of the copolymer metallocomplexes, the “direct copolymerization” method was also employed, as shown in route b of Scheme 2. For this approach, the bis-tridentate iridium complexes **C_12_Otpy-Ir-HOpy** and **CH_3_tpy-Ir-HOpy** were co-polymerized with the free 2,6-dihydroxyphenylpyridine (**HOpy**) and difluorophenyl-sulfone (**diFSO_2_**) to afford the copolymer metallocomplexes denoted as **b-CPOL-C_12_Otpy-Ir(x)** or **b-CPOL-CH_3_tpy-Ir(x)**. Since the molecular bis-tridentate iridium complexes bear two active hydroxyl groups on the diphenyl pyridine ligand, they could be readily polymerized using a condensation polymerization reaction. High temperature polyetherification conditions were employed in a high boiling point non-protic solvent (DMA) using potassium carbonate as the base. Toluene was also added to this polymerization medium to create an azeotropic mixture with the water formed during the polymerization and to aid the kinetics of the reaction.

For both the “post-polymerization” and “direct copolymerization” routes, up to 50 mol% of iridium loadings were tested. Regardless of the polymer metallocomplex synthetic method, namely route a or route b, efforts to increase further the bis-tridentate iridium complexes ratio did not provide higher iridium loadings despite the reaction time and temperature. This, however, is not considered as a disadvantage since in these copolymers, an additional purpose is served, namely that the uncomplexated diphenyl pyridine moieties act both as spacers between the complexes and hosts.

At this point, the choice of the difluoro-phenyl-sulfone (**diFSO_2_**) should be clarified; this was used as a comonomer in the synthesis of the uncomplexed homopolymer ligand (**pySO_2_**) and for the copolymer metallocomplex **CPOL-Rtpy-Ir(x)**. Though a big variety of aromatic difluorides is available, the particular difluoro-phenyl-sulfone is known to afford high molecular weight aromatic polyethers, as has been extensively described in previous works of our laboratory [52]. Additionally, it imposes excellent thermal, chemical, and oxidative stability to aromatic polyethers, and it improves the solubility of the final materials, thus allowing for processability and film formation via solution casting. Additionally, for the herein scope of creating soluble, processable, and light-emitting iridium metallopolymers, the insertion of sulfone moieties enhances the charge transporting properties of the polymeric metallocomplexes, as the sulfone moiety is known for its excellent electron-transporting properties [53].

A summary table of all polymer-Ir metallocomplexes prepared and studied in this work is given below (Table 1). The polymers are categorized based on the synthetic route that was employed, namely the “post-polymerization” complexation or the “direct copolymerization” routes.

### 3.2. Structural Characterization

The good solubility of the herein-synthesized molecular and polymer complexes in common organic solvents like CHCl_3_, THF, DMSO, and CH_3_CN allowed for the detailed characterization of their structural, optical, and electrochemical properties. Despite the high, in most cases, metal loadings, the highly soluble polymer backbone and the solubilizing side dodecyloxy or methyl chains on the terpyridine ligands assured the solubility of the metallocomplex monomers and copolymers.

**Figure 1:**
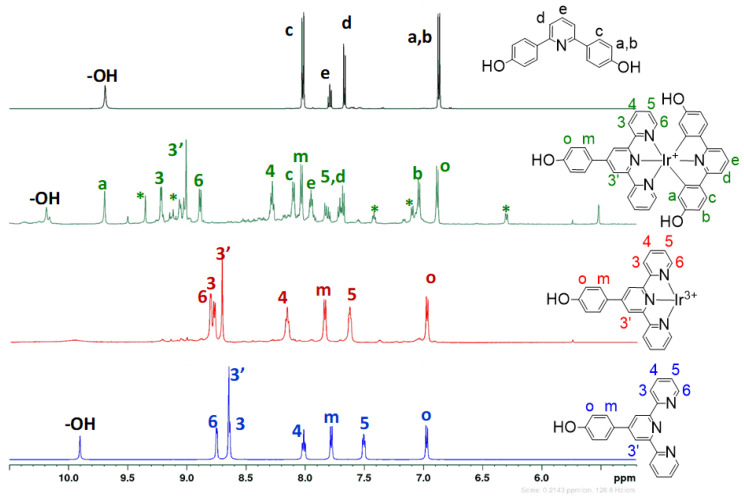
^1^H-NMR spectra of **HOtpy**, **HOtpy-IrCl_3_**, **HOtpy-Ir-HOpy**, and **HOpy** all in DMSO-d_6_. Peaks denoted with asterisks refer to alternative complexation modes or residual solvent traces. The area of 5 ppm to 0 ppm is provided in Figure S1.

Starting from the heteroleptic monomer complexes **Rtpy-Ir-HOpy**, their chemical structure was verified via ^1^H-NMR spectroscopy. Figure 1 and Figures S1–S3 present the ^1^H-NMR spectra of the ligands, along with the mono and dicomplexes for the three different terpyridines with a hydroxyl, a dodecyloxy, and a methyl group. The monocomplexes of the terpyridine ligands with IrCl_3_—**HOtpy-IrCl_3_**, **C_12_Otpy-IrCl_3_**, and **CH_3_tpy-IrCl_3_**—showed small but distinct downfield shifts of the nitrogen-containing phenyl ring proton signals (3, 3″, 4, 4″, 5, 5″, 6, and 6″) compared to the uncomplexated organic terpyridines. For the **HOtpy-IrCl_3_** and **CH_3_tpy-IrCl_3_** monocomplexes, unequivocally pure materials were obtained, as is evident in Figure 1, Figures S1 and S3. In contrast, the **C_12_Otpy-IrCl_3_** monocomplex (Figure S2) showed a more complicated spectrum in accordance with previous literature findings in which mixtures of the terpyridine-IrCl_3_ monocomplexes assigned to different structural motives have been reported [16,17,54]. However, in all those previous literature cases, the terpyridine-IrCl_3_ monocomplexes were used without any further purification. Therefore, in our case, we also proceeded with the preparation of the dicomplexes without the further purification of the **Rtpy-IrCl_3_** monocomplexes.

From the peak assignment of Figure 1, Figures S1 and S3, we can conclude that for the **HOtpy-Ir-HOpy** and **CH_3_tpy-Ir-HOpy** complexes, a (N^N^N)-(C^N^C) complexation mode was achieved. For **C_12_Otpy-Ir-HOpy**, as shown in Figure S2, the main percentage of the product also corresponded to the same complexation mode, although additional complexation modes were formed simultaneously, as was evident from the co-existence of additional peaks. With the incorporation of the first and then the second ligand, the NMR proton peaks were shifted downfield due to the coupling to the metal center. In particular, the superimposed peaks at 8.6 ppm of **HOtpy** in Figure 1 separated into two well-resolved peaks: a singlet, and a doublet at 8.78 and 8.7 ppm, respectively. In this case, the C4 proton, which could complexate with iridium, was still visible in the NMR spectrum and had the appropriate integration of 2. As a result, we can conclude that iridium formed an Ir(N^N^N) monocomplex with **HOtpy**. The dicomplex **HOtpy-Ir-HOpy** formed from **HOtpy-IrCl_3_** and **HOpy** was extensively purified using a series of solvents, and the respective ^1^H-NMR spectrum (Figure 1 and Figure S1 green spectrum) confirmed the successful synthesis, although some traces of unreacted starting monocomplex could not be entirely removed. Again, in the dicomplex, it was confirmed that once the Ir-N and Ir-C bonds were created, all protons were de-shielded because of the extended “spin-orbit” coupling (SOC) effect of the metal center; thus, all peaks shifted downfield with respect to the free ligands. When the hydroxyl group on the tpy ligand was changed to a dodecyloxy or a methyl group (**C_12_Otpy-Ir-HOpy, CH_3_tpy-Ir-HOpy**), the same conclusions could be drawn. The peaks were again shifted, indicating the successful desired complexation mode, as shown in Figures S2 and S3. ATR spectroscopy was also employed for the structural characterization of the complexes (Figure S10). The characteristic stretching vibration of the PF_6_^-^ ions at about 550 cm^−1^ was evident in the final complexes **C_12_Otpy-Ir-HOpy** and **CH_3_tpy-Ir-HOpy**.

In order to assess the successful synthesis of the target complexes, MALDI-TOF MS and LDI-TOF MS were employed. It is worth noting that for the monocomplexes **HOtpy-IrCl_3_**, **C_12_Otpy-IrCl_3_**, and **CH_3_tpy-IrCl_3_**, the spectra, in some cases, contained smaller fragments that corresponded to the bridged dimers that formed due to the laser ionization/fragmentation of the molecules while performing the measurements (Figures S4–S9).

**Figure 2:**
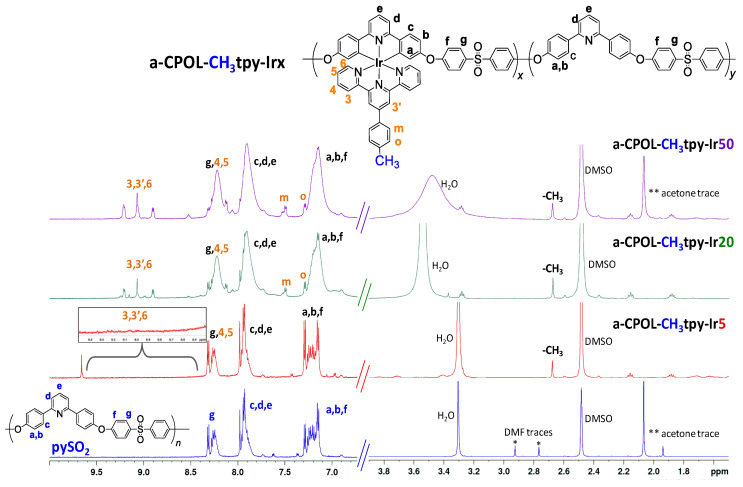
^1^H-NMR spectra of **a-CPOL-CH_3_tpy-Irx** (where **x = 5** red, **x = 20** green, and **x = 50** purple spectra) prepared by the “post-polymerization” complexation method and of the uncomplexated homopolymer ligand **pySO_2_** (blue), all in DMSO-d_6_. The inset shows a magnification of the 9.5–8.4 ppm area of the **a-CPOL-CH_3_tpy-Ir5** spectrum.

As expected, the polymeric iridium complexes provided even more complicated ^1^H-NMR spectra due to the high number of repeating units, and they also displayed broader peaks. Representative ^1^H NMR spectra of the polymer metallocomplexes **a-CPOL-CH_3_tpy-Irx** prepared by the “post-polymerization” complexation method are presented in Figure 2. The spectrum of the parent homopolymer, uncomplexated polymer ligand **pySO_2_**, is also included. The spectra are dominated by the peaks of the uncomplexated diphenyl pyridine moieties, as these were in larger percentages in most cases. From the spectra of **a-CPOL-CH_3_tpy-Ir20**, **a-CPOL-CH_3_tpy-Ir50** (Figure 2), and **a-CPOL-C_12_Otpy-Ir50** (Figure S11), the actual metal loadings were calculated using the integration of the peaks at 9.2, 9.07, or 8.9 ppm, that were solely attributed to the terpyridine groups versus the polymer backbone aromatic protons at 8.4–6.8 ppm. These were found equal to 10% metal loading for **a-CPOL-CH_3_tpy-Ir20**, 21% metal loading for **a-CPOL-CH_3_tpy-Ir50**, and 18% metal loading for **a-CPOL-C_12_Otpy-Ir50**. Unfortunately, the rest of the copolymer metallocomplexes did not present so distinctively clear ^1^H NMR spectra, and the terpyridine aromatic proton peaks were hardly evident in the spectra; however, this did not comply with the samples’ reddish color or the optoelectronic properties of the materials, as is discussed below. In agreement with the ATR results of the monomeric Ir complexes, the iridium metallopolymers showed the characteristic stretching vibration of the PF_6_^−^ ions at about 550 cm^−1^ in their ATR spectra, with no other significant differentiation in comparison to the initial homopolymer macroligand **pySO_2_** and regardless of the complexation degree (Figure S12).

**Figure 3:**
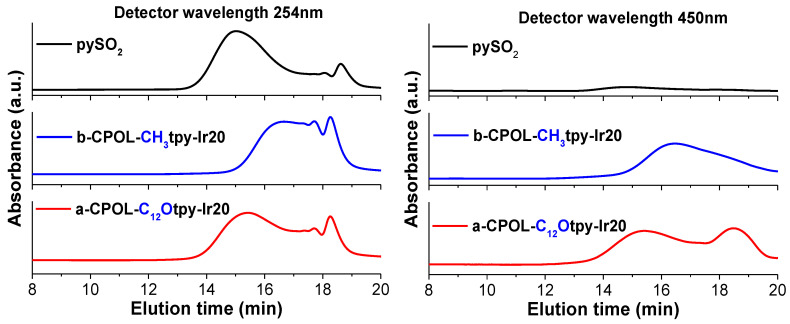
Gel permeation chromatography (GPC) traces of the parent polymer **pySO_2_** (black) and two representative metallopolymers **b-CPOL-CH_3_tpy-Ir20** (blue), and **a-CPOL-C_12_Otpy-Ir20** (red). The UV detector was set at 254 nm (left curves) or at 450 nm (right curves).

The molecular weights of the synthesized polymeric metallocomplexes were assessed by gel permeation chromatography (GPC) using CHCl_3_ as the eluting solvent (Table 2). Representative chromatograms are shown in Figure 3 and Figure S13. The UV detector was set at 254 or at 450 nm, at which wavelengths the organic moieties and the tpy-Ir-py complexes absorb, respectively. Through this way, the uncomplexated homopolymer ligand (**pySO_2_**) could be distinguished from the polymer metallocomplexes. In the absence of tpy-Ir-py complexes along the polymer backbone, detection at 450 nm did not afford a signal since the neat homopolymer does not absorb at this wavelength (Figure 3). It should also be pointed out that in some cases, the metallocopolymers prepared via route a showed lower molecular weights compared to the initial homopolymer **pySO_2_**, as only the lower molecular weight fractions were soluble in CHCl_3_ due to the large metal loadings.

### 3.3. Photophysical Properties

**Figure 4:**
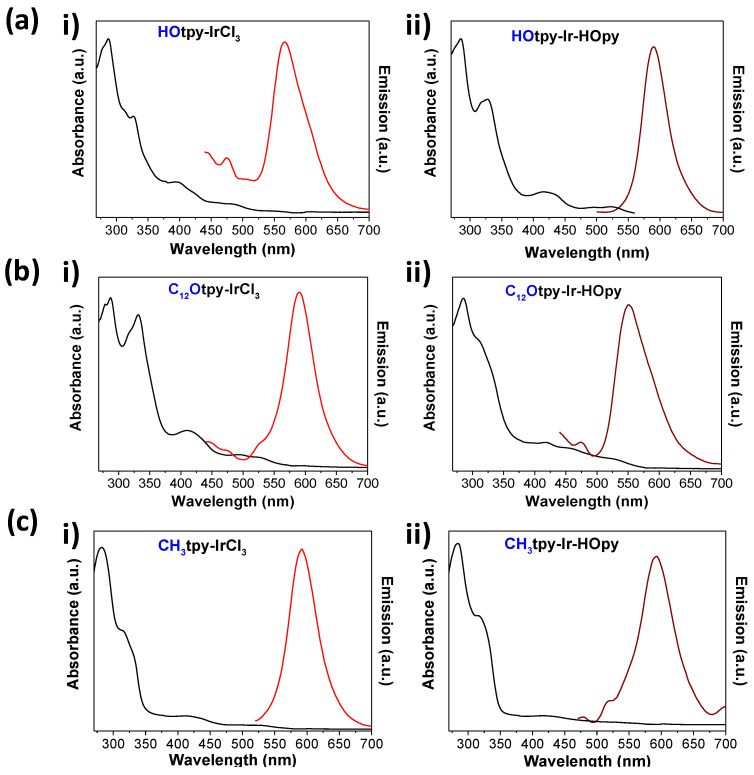
Absorption (black) and emission (colored) spectra in DMF solution of (**a**) (**i**) **HOtpy-IrCl_3_** and (**ii**) **HOtpy-Ir-HOpy**; (**b**) (**i**) **C_12_Otpy-IrCl_3_** and (**ii**) **C_12_Otpy-Ir-HOpy**; and (**c**) (**i**) **CH_3_tpy-IrCl_3_** and (**ii**) **CH_3_tpy-Ir-HOpy**.

The UV–vis absorption and emission spectra of the synthesized complexes were measured in different solvents at room temperature, and the data are presented in Table 3. All complexes exhibited similar behavior with bands <400 nm corresponding to π–π* ligand-centered transitions, while all metal-ion-containing compounds had two absorption bands at ~420 and ~500 nm that are characteristic absorption bands of iridium complexes, being ascribed to ^1^MLCT and ^3^MLCT states. In particular, **HOtpy-IrCl_3_** and **HOtpy-Ir-HOpy** (Figure 4a) exhibited similar absorption bands at 300 and 327 nm, respectively, with the first absorption band being ascribed to ^1^LC states and the second absorption band being ascribed to ^3^LC states. The two bands at lower energies are characteristic absorption bands for iridium complexes that are, respectively, attributed to ^1^MLCT and ^3^MLCT states. Meanwhile, the monocomplex emitted at 566 nm (Figure 4(ai)), and the fully complexated moiety emitted at lower energies at around 590 nm (Figure 4(aii)). The dodecyloxy group on the terpyridine ligand led to a slight difference in the absorption and emission profiles (Figure 4b) because the dodecyloxy group is a stronger donor than HO- and therefore further strengthens the SOC effect of the metal. The methyl terpyridine functionalized complexes behaved much in the same way as their dodecyloxy counterparts, having similar emission maxima. All characteristic emissions at about 590 nm were broad and featureless, in agreement with previous results for Ir(N^N^N)(C^N^C) complexes [16,17,32]. From the photophysical data presented herein, it can be deduced that all synthesized monomeric complexes emitted in the orange–red region of the visible spectrum.

**Figure 5:**
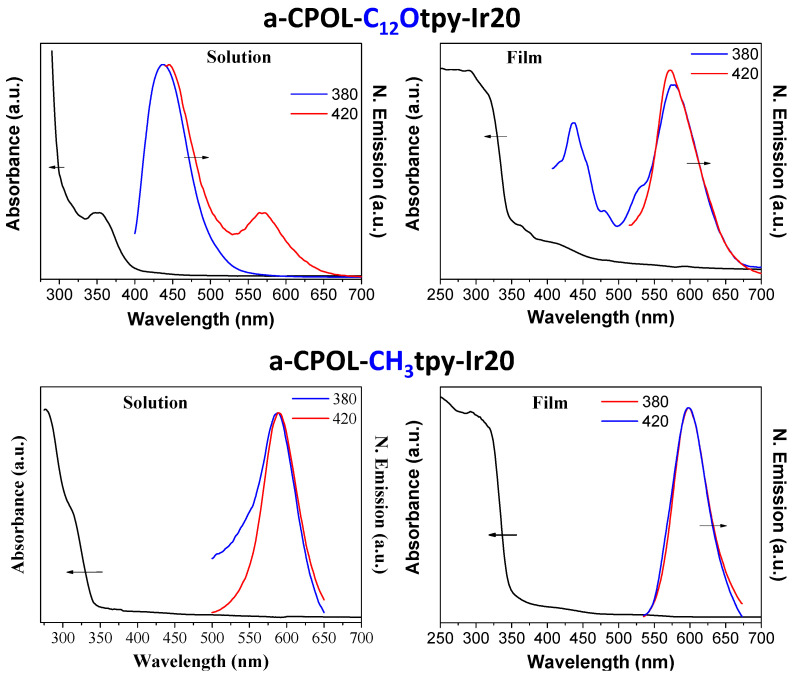
Absorption (black) and normalized emission (colored) spectra of **a-CPOL-Rtpy-Ir20** in 1,1′,2,2′,-TCE solutions (left) and in film form (right) at two different excitation wavelengths.

The copolymeric complexes displayed similar behavior to their monomeric counterparts, with absorption maxima at around 270 or 290 nm and an ^1^MLCT band at 415 nm. However, the ^3^MLCT absorption band strongly depended on the metal loading of the metallocopolymer and, in many cases, was barely visible (Figure 5, Figures S14 and S15) for both the solutions and the thin films of the copolymers. The UV–vis and PL spectra of the initial homopolymer **pySO_2_** in solution and in thin film form are provided in Figure S16, showing emission maxima at about 350 and 400 nm in solution and film, respectively, as expected for purely organic materials without the presence of extended conjugation. On the other hand, all metallocopolymers presented the characteristic emission bands of the iridium complexes at 550–600 nm.

### 3.4. Electrochemical Properties

**Figure 6:**
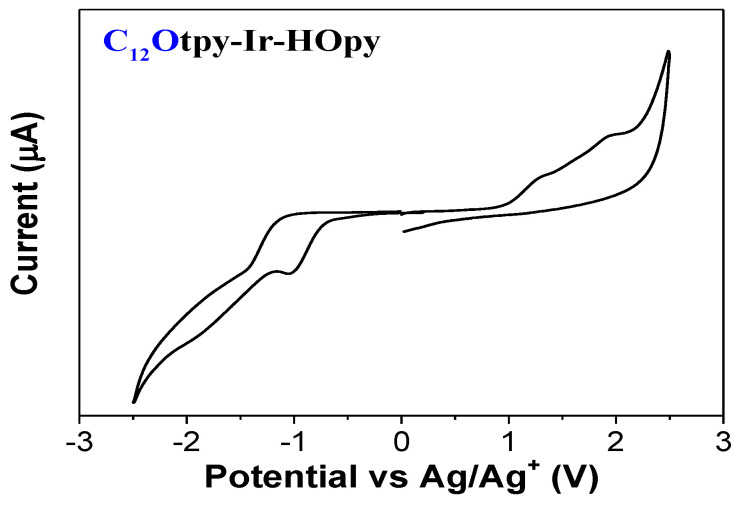
Cyclic voltammogram of **C_12_Otpy-Ir-HOpy** recorded in acetonitrile with a scan rate of 100 mV/sec and ferrocene as the internal standard.

In order to determine the energy levels (HOMO and LUMO levels) of the complexes, cyclic voltammograms were recorded for thin films deposited onto an ITO glass substrate, and a representative voltammogram is provided in Figure 6. The **C_12_Otpy-Ir-HOpy** molecular complex displayed one non-reversible peak at −1.05 V that corresponded to the reduction of the ligand and another non-reversible peak at 1.32 V. The oxidation wave also had a second peak at around 2 V. All peaks were non-reversible. Using the general equations for the HOMO and LUMO levels, we could calculate the approximate energies of the HOMO and LUMO orbitals of the complex. As a result, a HOMO = −5.76 eV and a LUMO = −3.72 eV were estimated. These results indicate that the molecular iridium complexes are suitable for incorporation in EMLs with traditional hosts such as poly(9-vinylcarbazole) (PVK).

**Figure 7:**
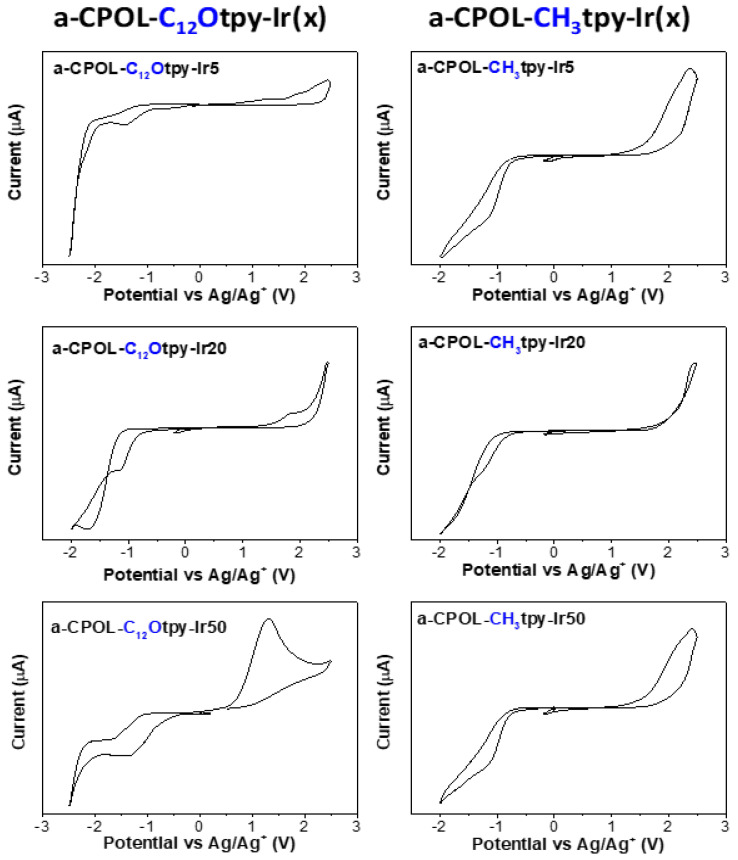
Cyclic voltammograms of metallocopolymers **a-CPOL-C_12_Otpy-Irx** and **a-CPOL-CH_3_tpy-Irx** recorded in acetonitrile with a scan rate of 100mV/sec and ferrocene as the internal standard.

The cyclic voltammograms of representative polymeric metallocomplexes are presented in Figure 7. The **a-CPOL-C_12_Otpy-Ir50**, for example, displayed an irreversible peak at around −1.35 V, while at the anodic wave, it displayed a peak at 2.0 V. Using the same calculation method as for **C_12_Otpy-Ir-HOpy**, the HOMO and LUMO energies were estimated to be almost the same as those of the molecular complex at −5.36 and −4.03 V, respectively. This could be attributed to the high metal loading of the copolymer that essentially led to the same electrochemical characteristics for the metallocopolymer as for the molecular complex.

**Figure 8:**
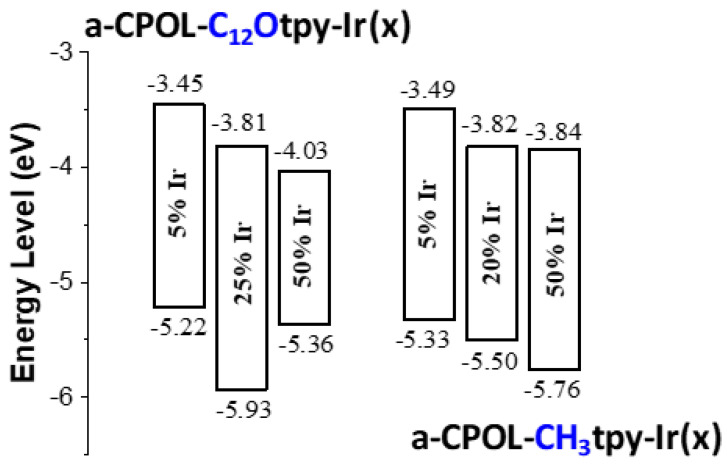
Energy levels of a-CPOL-C_12_Otpy-Irx (left) and a-CPOL-CH_3_tpy-Irx (right).

A schematic diagram of all copolymeric metallocomplexes energy levels is presented in Figure 8. Notably, in the case of the polymeric metallocomplexes, the different metal loadings had direct effects on their energy levels. The LUMO levels showed a disparity of up to 0.58 eV for **a-CPOL-C_12_Otpy-Irx** when the metal loading increased from 5% to 50%, while the **a-CPOL-CH_3_tpy-Irx** metallopolymers showed a smaller difference of about 0.3 eV (Figure 8). This behavior was expected since greater metal loadings destabilize the orbital of the molecule. The difference of the energy levels for the 20% and the 50% metal-loaded copolymers was not as great as in the case of the smaller loadings. As far as the HOMO levels are concerned, the differences were smaller between the different metallopolymers since the HOMO is mainly centered on the Ir-C^N^C part of the complex [17]; therefore, the HOMO is mainly affected by the polymeric chain and not by the complexation degree.

## 4. Conclusions

In summary, new monomeric and polymeric bis-tridendate iridium complexes of the coordination scheme Ir(N^N^N)(C^N^C) were successfully synthesized and characterized in this study. Starting from monomeric terpyridine-iridium-diphenylpyridine complexes, these were decorated with hydroxyl-, dodecyloxy-, or methyl-groups at the fourth position of the phenyl-terpyridine ring, thus enhancing their solubility in common organic solvents. All monomer-Ir-complexes were structurally characterized using ^1^H-NMR and ATR spectroscopies, while their optical properties evaluation revealed that they emitted in the orange–red region of the visible spectrum with an emission maximum of around 580–600 nm. Furthermore, the energy levels for the dodecyloxy-bearing complex were determined in the solid state using cyclic voltammetry.

These monomeric-Ir-complexes were the predecessors of our work’s main target, which was solution-processable polymer-Ir-metallocomplex phosphors of the Ir(N^N^N)(C^N^C) coordination scheme. In order to ascertain whether these herein-developed Ir-complexes could be successfully incorporated along polymer chains, two strategies were used. The first strategy involved the “post-polymerization complexation” of a homopolymer bearing diphenyl-pyridine main chain moieties, acting as a macromolecular ligand. The second strategy, “direct copolymerization,” involved the copolymerization of the bis-cyclometalated iridium monomeric complexes bearing dodecyloxy- or methyl-solubilizing chains with the co-monomers dihydroxyl-phenyl-pyridine and bis-(4-fluorophenyl)-sulfone. Both cases afforded hybrid bis-tridendate Ir(III) polymer-metallocomplexes of good solubility in common organic solvents, thus allowing for their thorough structural and optoelectronic characterization. The synthesized polymeric complexes showed emission around 590 nm in film form. The energy levels of the hybrid Ir-metallopolymers, as assessed by CV measurements in film form, revealed a dependence of their LUMO levels on the Ir loading of the polymers. The polymer-Ir complexes of higher Ir loadings presented lower LUMO levels compared to the lower Ir-loaded polymers.

Evidently, in this study, rare iridium small molecular and polymeric metallocomplexes were successfully developed, thus showing the potentiality of the herein-presented synthetic route toward soluble, processable, and film-forming hybrid iridium metallopolymers of narrow red-light emission for OLED applications.

## Figures and Tables

**Table 1:** Polymer metallocomplexes synthesized in this study.

Code	Ir (%) ^a^	Synthetic Method	Solubility ^b,c^	
**“Post-Polymerization” Complexation** **a-CPOL-Rtpy-Ir(x)**				
**a-CPOL-C_12_Otpy-Ir5**	5	Post-complexation	TCE	✓✓✓
**a-CPOL-C_12_Otpy-Ir20**	20	Post-complexation	TCE	✓✓✓
**a-CPOL-C_12_Otpy-Ir50**	50	Post-complexation	CHCl_3_	✓
			DMSO, NMP	✓✓
			DMF, DMA, TCE	✓✓✓
**a-CPOL-CH_3_tpy-Ir5**	5	Post-complexation	TCE	
**a-CPOL-CH_3_tpy-Ir20**	20	Post-complexation	CHCl_3_	✓
			DMSO, NMP	✓✓
			DMF, DMA, TCE	✓✓✓
**a-CPOL-CH_3_tpy-Ir50**	50	Post-complexation	CHCl_3_	✓
			DMSO, NMP	✓✓
			DMF, DMA, TCE	✓✓✓
**“Direct copolymerization”** **b-CPOL-Rtpy-Ir(x)**				
**b-CPOL-C_12_Otpy-Ir5**	5	Co-polymerization	TCE	✓✓✓
**b-CPOL-C_12_Otpy-Ir25**	25	Co-polymerization	CHCl_3_	✓
			DMSO, NMP	✓✓
			DMF, DMA, TCE	✓✓✓
**b-CPOL-C_12_Otpy-Ir50**	50	Co-polymerization	CHCl_3_,	✓
			NMP, DMSO	✓✓
			TCE, DMF, DMA	✓✓✓
**b-CPOL-CH_3_tpy-Ir5**	5	Co-polymerization	TCE	✓✓✓
**b-CPOL-CH_3_tpy-Ir20**	20	Co-polymerization	CHCl_3_,	✓
			NMP, DMSO	✓✓
			TCE, DMF, DMA	✓✓✓
**b-CPOL-CH_3_tpy-Ir50**	50	Co-polymerization	CHCl_3_,	✓
			NMP, DMSO	✓✓
			TCE, DMF, DMA	✓✓✓

^a^ Theoretical Ir(III) molar percentage based on the feed ratio of the reaction. ^b^ Solubility tests were performed at ambient conditions (25 °C), unless otherwise specified, using 1wt% solutions. ^c^ ✓ = low solubility (less than 0.5% *w/v*); ✓✓ = partially soluble (1–2% *w/v*); ✓✓✓ = fully soluble (higher than 2% *w/v*).

**Table 2:** Molecular characteristics of the polymeric metallocomplexes ^a^.

Polymer	Mn ^b^	Mw ^b^	PD ^b^
**pySO_2_**	16,400	27,500	1.7
**a-CPOL-C_12_Otpy-Ir5**	-	-	-
**a-CPOL-C_12_Otpy-Ir20**	15,640	30,470	1.9
**a-CPOL-C_12_Otpy-Ir50**	4070	6660	1.6
**b-CPOL-C_12_Otpy-Ir5**	7400	17,330	2.3
**b-CPOL-C_12_Otpy-Ir20**	4360	6790	1.6
**b-CPOL-C_12_Otpy-Ir50**	13,900	32,000	2.3
**a-CPOL-CH_3_tpy-Ir5**	11,480	25,370	2.2
**a-CPOL-CH_3_tpy-Ir20**	11,100	19,550	1.8
**a-CPOL-CH_3_tpy-Ir50**	7200	9500	1.3
**b-CPOL-CH_3_tpy-Ir5**	9230	17,100	1.8
**b-CPOL-CH_3_tpy-Ir20**	7780	12,130	1.6
**b-CPOL-CH_3_tpy-Ir50**	4600	6600	1.5

^a^ Chromatograms were obtained at 25 °C using chloroform as the eluent versus PS standards. The UV detector was set at 254 nm. ^b^ Mn = number average molecular weight; Mw = mass average molecular weight; and PD = polydispersity.

**Table 3:** Photophysical data of the compounds synthesized in the study.

Compound	Absorption (nm)	Emission (nm)	Medium
**HO** **tpy-IrCl_3_**	287, 327, 401, and 490	566	DMF
**HOtpy-Ir-HOpy**	288, 328, 415, and 523	590	DMF
**C_12_Otpy-IrCl_3_**	287, 332, 410, 494, and 526	589	DMF
**C_12_Otpy-Ir-HOpy**	285, 312 (shoulder), 418, 457, and 525	594	DMF
**CH_3_tpy-IrCl_3_**	282, 317 (shoulder), 418, and 520	592	DMF
**CH_3_tpy-Ir-HOpy**	285, 317, and 419	592 and 705	DMF
**pySO_2_**			
**a-CPOL-C_12_Otpy-Ir20**	353	437 and 571	TCE
**a-CPOL-C_12_Otpy-Ir20**	290 and 415	438 and 573	Film
**a-CPOL-CH_3_tpy-Ir20**	278 and 311	590	TCE
**a-CPOL-CH_3_tpy-Ir20**	237 and 293	600	Film

## Supplementary Materials

The following are available online at https://www.mdpi.com/2073-4360/12/12/2976/s1, Figure S1. ^1^H-NMR spectra area of 5 ppm to 0 ppm of **HO****tpy**, **HO****tpy-IrCl_3_**, **HOtpy-Ir-HOpy** and **HOpy** all in DMSO-d_6_. Figure S2. ^1^H-NMR spectra of **C_12_Otpy** in CDCl_3_, **C_12_Otpy-IrCl_3_** in DMSO-d_6_, and **C_12_Otpy-Ir-HOpy** in DMSO-d_6_. Figure S3. ^1^H-NMR spectra of **CH_3_tpy** in CDCl_3_, **CH_3_tpy-IrCl_3_** in DMSO-d_6_, and **CH_3_tpy-Ir-HOpy** in DMSO-d_6_. Figure S4. LDI – TOF/MS spectra of **HO****tpy-IrCl_3_**. Figure S5. LDI–TOF/MS spectra of **C_12_Otpy-IrCl_3_**. Figure S6. LDI–TOF/MS spectra of **CH_3_tpy-IrCl_3_**. Figure S7. LDI–TOF/MS spectra of **HOtpy-Ir-HOpy**. Figure S8. LDI-TOF MS spectra of **C_12_Otpy-Ir-HOpy**. Figure S9. LDI–TOF/MS spectra of **CH_3_tpy-Ir-HOpy.** Figure S10. ATR spectra of the monomeric Ir(III) complexes based on the **C_12_Otpy** and **CH_3_tpy** ligands. Figure S11. ^1^H-NMR spectra of **a-CPOL-C_12_Otpy-Irx** (where x = 20% red and x = 50% green) prepared by the “post polymerization” complexation method, and of the uncomplexed homopolymer ligand **pySO_2_** in DMSO-d_6_. (a) shows the region of 10.6 ppm and (b) the region of 3.0 ppm. The inset in (a) shows a magnification of the 10–8.5ppm area of the **a-CPOL-C_12_Otpy-Ir20** spectrum. Figure S12. ATR spectra of the polymeric Ir(III) complexes **b-CPOL-CH_3_tpy-Irx**. Figure S13. GPC trace of **a-CPOL-Rtpy-Irx** (indicated as **a-R-Irx**) and **b-CPOL-Rtpy-Irx** (indicated as **b-R-Irx**) with the detector set at 254 nm (left) and 450 nm (right). Figure S14. Absorption (black) and emission (color) spectra of the polymeric complex **a-CPOL-C_12_Otpy-Irx**, where x = 5 and 50 recorded in a 1,1′,2,2′-TCE solution (left) and thin film (right). Figure S15. Absorption (black) and emission (colored) spectra of the polymeric complex **a-CPOL-CH_3_tpy-Irx**, where x = 5 and 50 recorded in 1,1′,2,2′-TCE solution (left) and thin film (right). Figure S16. Absorption and emission spectra of **pySO_2_** in (a) solution using DMA and in (b) thin film form.

## References

[1] Baldo M.A., You Y., O’Brien D.F., Shoustikov A., Sibley S., Thompson M.E., Forrest S.R. (1998). Highly efficient phosphorescent emission from organic electroluminescent devices. Nature.

[2] Ma D., Tsuboi T., Qiu Y., Duan L. (2017). Recent progress in ionic iridium(III) complexes for organic electronic devices. Adv. Mater..

[3] Mao H.T., Li G.F., Shan G.G., Wang X.L., Su Z.M. (2020). Recent progress in phosphorescent Ir(III) complexes for nondoped organic light-emitting diodes. Coord. Chem. Rev..

[4] Henwood A.F., Zysman-Colman E. (2016). Luminescent iridium complexes used in light-emitting electrochemical cells (LEECs). Top. Curr. Chem..

[5] Namanga J.E., Pei H., Bousrez G., Mallick B., Smetana V., Gerlitzki N., Mudring A.V. (2020). Efficient and long lived green light-emitting electrochemical cells. Adv. Funct. Mater..

[6] Caporale C., Massi M. (2018). Cyclometalated iridium(III) complexes for life science. Coord. Chem. Rev..

[7] Glaser F., Wenger O.S. (2020). Recent progress in the development of transition-metal based photoredox catalysts. Coord. Chem. Rev..

[8] Wang T., Sun R., Shi M., Pan F., Hu Z., Huang F., Li Y., Min J. (2020). Solution-processed polymer solar cells with over 17% efficiency enabled by an iridium complexation approach. Adv. Energy Mater..

[9] Housecroft C.E., King R.B., Crabtree R.H., Lukehart C.M., Atwood D.A., Scott R.A. (2006). Iridium: Inorganic & coordination chemistry. Encyclopedia of Inorganic Chemistry.

[10] Yang H., Meng G., Zhou Y., Tang H., Zhao J., Wang Z. (2015). The photoluminescent properties of new cationic iridium(III) complexes using different anions and their applications in white light-emitting diodes. Materials.

[11] Tang H., Meng G., Chen Z., Wang K., Zhou Q., Wang Z. (2017). Warm white light-emitting diodes based on a novel orange cationic iridium(III) complex. Materials.

[12] Wang S., Zhang H., Zhang B., Xie Z., Wong W.Y. (2020). Towards high-power-efficiency solution-processed OLEDs: Material and device perspectives. Mater. Sci. Eng. R Rep..

[13] Adeloye A.O. (2019). Exploration of the structural and photophysical characteristics of mono- and binuclear Ir (III) cyclometalated complexes for optoelectronic applications. Materials.

[14] Baranoff E., Curchod B.F.E. (2015). FIrpic: Archetypal blue phosphorescent emitter for electroluminescence. Dalt. Trans..

[15] Wu S., Li S., Sun Q., Huang C., Fung M.K. (2016). Highly efficient white organic light-emitting diodes with ultrathin emissive layers and a spacer-free structure. Sci. Rep..

[16] Collin J.P., Dixon I.M., Sauvage J.P., Williams J.A.G., Barigelletti F., Flamigni L. (1999). Synthesis and photophysical properties of iridium(III) bisterpyridine and its homologues: A family of complexes with a long-lived excited state. J. Am. Chem. Soc..

[17] Polson M., Fracasso S., Bertolasi V., Ravaglia M., Scandola F. (2004). Iridium cyclometalated complexes with axial symmetry. Synthesis and photophysical properties of a trans-biscyclometalated complex containing the terdentate ligand 2,6-diphenylpyridine. Inorg. Chem..

[18] Williams J.A.G., Wilkinson A.J., Whittle V.L. (2008). Light-emitting iridium complexes with tridentate ligands. J. Chem. Soc. Dalt. Trans..

[19] Chi Y., Chang T.K., Ganesan P., Rajakannu P. (2017). Emissive bis-tridentate Ir(III) metal complexes: Tactics, photophysics and applications. Coord. Chem. Rev..

[20] Goodall W., Williams J.A.G. (2000). Iridium(III) bis-terpyridine complexes incorporating pendent YV-methylpyridinium groups: Luminescent sensors for chloride ions. J. Chem. Soc. Dalt. Trans..

[21] Whittle V.L., Williams J.A.G. (2008). A new class of iridium complexes suitable for stepwise incorporation into linear assemblies: Synthesis, electrochemistry, and luminescence. Inorg. Chem..

[22] Agosti A., Kuna E., Bergamini G. (2019). Divergent terpyridine-based coordination for the construction of photoactive supramolecular structures. Eur. J. Inorg. Chem..

[23] Liu B., Monro S., Li Z., Jabed M.A., Ramirez D., Cameron C.G., Colón K., Roque J., Kilina S., Tian J. (2019). New class of homoleptic and heteroleptic bis(terpyridine) iridium(III) complexes with strong photodynamic therapy effects. ACS Appl. Bio Mater..

[24] Darmawan N., Yang C.H., Mauro M., Raynal M., Heun S., Pan J., Buchholz H., Braunstein P., de Cola L. (2013). Efficient near-UV emitters based on cationic bis-pincer iridium(III) carbene complexes. Inorg. Chem..

[25] Lin J., Chau N.Y., Liao J.L., Wong W.Y., Lu C.Y., Sie Z.T., Chang C.H., Fox M.A., Low P.J., Lee G.H. (2016). Bis-tridentate iridium(III) phosphors bearing functional 2-phenyl-6-(imidazol-2-ylidene)pyridine and 2-(pyrazol-3-Yl)-6-phenylpyridine chelates for efficient OLEDs. Organometallics.

[26] Kuo H.H., Chen Y.T., Devereux L.R., Wu C.C., Fox M.A., Kuei C.Y., Chi Y., Lee G.H. (2017). Bis-tridentate Ir(III) metal phosphors for efficient deep-blue organic light-emitting diodes. Adv. Mater..

[27] Chen Y.K., Kuo H.H., Luo D., Lai Y.N., Li W.C., Chang C.H., Escudero D., Jen A.K.Y., Hsu L.Y., Chi Y. (2019). Phenyl-A Nd pyrazolyl-functionalized pyrimidine: Versatile chromophore of bis-tridentate Ir(III) phosphors for organic light-emitting diodes. Chem. Mater..

[28] Wilkinson A.J., Goeta A.E., Foster C.E., Williams J.A.G. (2004). Synthesis and luminescence of a charge-neutral, cyclometalated iridium(III) complex containing N/\C/\N- and C/\N/\C-coordinating terdentate ligands. Inorg. Chem..

[29] Adamovich V., Boudreault P.L.T., Esteruelas M.A., Gómez-Bautista D., López A.M., Oñate E., Tsai J.Y. (2019). Preparation via a NHC dimer complex, photophysical properties, and device performance of heteroleptic bis(tridentate) iridium(III) emitters. Organometallics.

[30] Hu Y.Y., Luo W., Wang Y., Tong B.H., Fung M.K., Zhang Q.F. (2020). Efficient yellow OLEDs based on bis-tridentate iridium(III) complexes with two C∧N∧N-coordinating ligands. Inorg. Chim. Acta.

[31] Kuo H.H., Zhu Z., Lee C.S., Chen Y.K., Liu S.H., Chou P.T., Jen A.K.Y., Chi Y. (2018). Bis-tridentate iridium(III) phosphors with very high photostability and fabrication of blue-emitting OLEDs. Adv. Sci..

[32] Kuei C.Y., Tsai W.L., Tong B., Jiao M., Lee W.K., Chi Y., Wu C.C., Liu S.H., Lee G.H., Chou P.T. (2016). Bis-tridentate Ir(III) complexes with nearly unitary RGB phosphorescence and organic light-emitting diodes with external quantum efficiency exceeding 31%. Adv. Mater..

[33] Andreopoulou A.K., Gioti M., Kallitsis J.K., Ulanski J., Luszczynska M., Matyjaszewski K. (2019). Organic light-emitting diodes based on solution-processable organic materials. Solution-Processable Components for Organic Electronic Devices.

[34] Chen X., Liao J.L., Liang Y., Ahmed M.O., Tseng H.E., Chen S.A. (2003). High-efficiency red-light emission from polyfluorenes grafted with cyclometalated iridium complexes and charge transport moiety. J. Am. Chem. Soc..

[35] Baschieri A., Monti F., Armaroli N., Mazzotti G., Giorgini L., Sambri L., Benelli T. (2019). Luminescent methacrylic copolymers with side-chain cyclometalated iridium(III) complexes. Dye. Pigment..

[36] Xu F., Kim H.U., Kim J.H., Jung B.J., Grimsdale A.C., Hwang D.H. (2015). Progress and perspective of iridium-containing phosphorescent polymers for light-emitting diodes. Progress in Polymer Science.

[37] Tang H., Dong X., Chen M., Chen Q., Ren M., Wang K., Zhou Q., Wang Z. (2019). A novel polymethyl methacrylate derivative grafted with cationic iridium(III) complex units: Synthesis and application in white light-emitting diodes. Polymers.

[38] Page Z.A., Chiu C.Y., Narupai B., Laitar D.S., Mukhopadhyay S., Sokolov A., Hudson Z.M., Bou Zerdan R., McGrath A.J., Kramer J.W. (2017). Highly photoluminescent nonconjugated polymers for single-layer light emitting diodes. ACS Photonics.

[39] Liu B., Dang F., Tian Z., Feng Z., Jin D. (2017). High triplet energy-level achieved by tuning the arrangement of building blocks in phosphorescent polymer backbones for furnishing high electroluminescent performances in both blue and white organic light-emitting devices high triplet energy-level achieve. ACS Appl. Mater. Interfaces.

[40] Aamer K.A., Tew G.N. (2007). Synthesis, dynamic light scattering, and luminescence properties of copolymers containing iridium(III) bisterpyridine in the side chain. J. Polym. Sci. Part A Polym. Chem..

[41] Zhen H., Jiang C., Yang W., Jiang J., Huang F., Cao Y. (2005). Synthesis and properties of electrophosphorescent chelating polymers with iridium complexes in the conjugated backbone. Chem. A Eur. J..

[42] Schulz G.L., Chen X., Chen S.A., Holdcroft S. (2006). Enhancement of phosphorescence of Ir complexes bound to conjugated polymers: Increasing the triplet level of the main chain. Macromolecules.

[43] Liu J., Pei Q. (2010). Electrophosphorescent polymers for high-efficiency light-emitting diodes. Curr. Org. Chem..

[44] Langecker J., Rehahn M. (2008). Iridium-functionalized polyfluorenes: Advantages and limitations of Thes Suzuki and Yamamoto approaches. Macromol. Chem. Phys..

[45] Huang S.P., Jen T.H., Chen Y.C., Hsiao A.E., Yin S.H., Chen H.Y., Chen S.A. (2008). Effective shielding of triplet energy transfer to conjugated polymer by its dense side chains from phosphor dopant for highly efficient electrophosphorescence. J. Am. Chem. Soc..

[46] Hammond M.R., Andreopoulou A.K., Pefkianakis E., Kallitsis J.K., Mezzenga R. (2009). Metallosupramolecular side-chain polymers and polyelectrolyte- metallosupramolecular surfactant complexes. Chem. Mater..

[47] Gourdoupi N., Andreopoulou A.K., Deimede V., Kallitsis J.K. (2003). Novel proton-conducting polyelectrolyte composed of an aromatic polyether containing main-chain pyridine units for fuel cell applications. Chem. Mater..

[48] Koromilas N.D., Anastasopoulos C., Oikonomou E.K., Kallitsis J.K. (2019). Preparation of porous polymeric membranes based on a pyridine containing aromatic polyether sulfone. Polymers.

[49] Heller M., Schubert U.S. (2003). Syntheses of functionalized 2,2′:6′,2″-terpyridines. Eur. J. Org. Chem..

[50] Constable E.C., Harverson P., Smith D.R., Whall L. (1997). The coordination chemistry of 4′-(4-tert-butylphenyl)-2,2′:6′,2″-terpyridine—A solubilising oligopyridine. Polyhedron.

[51] Andreopoulou A.K., Kallitsis J.K. (2005). An “Attachment through coordination” approach to side chain dendritic polymers. European J. Org. Chem..

[52] Kallitsis J.K., Andreopoulou A.K., Daletou M., Neophytides S., Li Q., Aili D., Hjuler H., Jensen J. (2016). Pyridine Containing Aromatic Polyether Membranes.

[53] Sasabe H., Seino Y., Kimura M., Kido J. (2012). A m-terphenyl-modifed sulfone derivative as a host material for high-efficiency blue and green phosphorescent OLEDs. Chem. Mater..

[54] Dixon I.M., Collin J.P., Sauvage J.P., Flamigni L., Encinas Perea S., Barigelletti F. (2000). A family of luminescent coordination compounds: Iridium(III) polyimine complexes. Chem. Soc. Rev..

