# Supplementary material for: Bis-Tridendate Ir(III) Polymer-Metallocomplexes: Hybrid, Main-Chain Polymer Phosphors for Orange–Red Light Emission
Source: Polymers (Basel). 2020 Dec 13;12(12):2976. doi: 10.3390/polym12122976 (PMC7764732; doi:10.3390/polym12122976)
Supplement: Supplementary file 1 [file polymers-12-02976-s001.pdf]

polymers

MDPI

Supporting Information

Bis-Tridendate Ir(III) Polymer-Metallocomplexes: Hybrid, Main-Chain Polymer Phosphors for Orange – Red Light Emission

K. Andrikopoulos[1], C. Anastasopoulos[1], J. K. Kallitsis[1,2], A. K. Andreopoulou[1,2,*]

[1] *Department of Chemistry, University of Patras, University Campus, Rio-Patras, GR26504, Greece*

[2] *Foundation for Research and Technology Hellas/Institute of Chemical Engineering Sciences (FORTH/ICE-HT), Platani Str., Patras, GR26504, Greece*

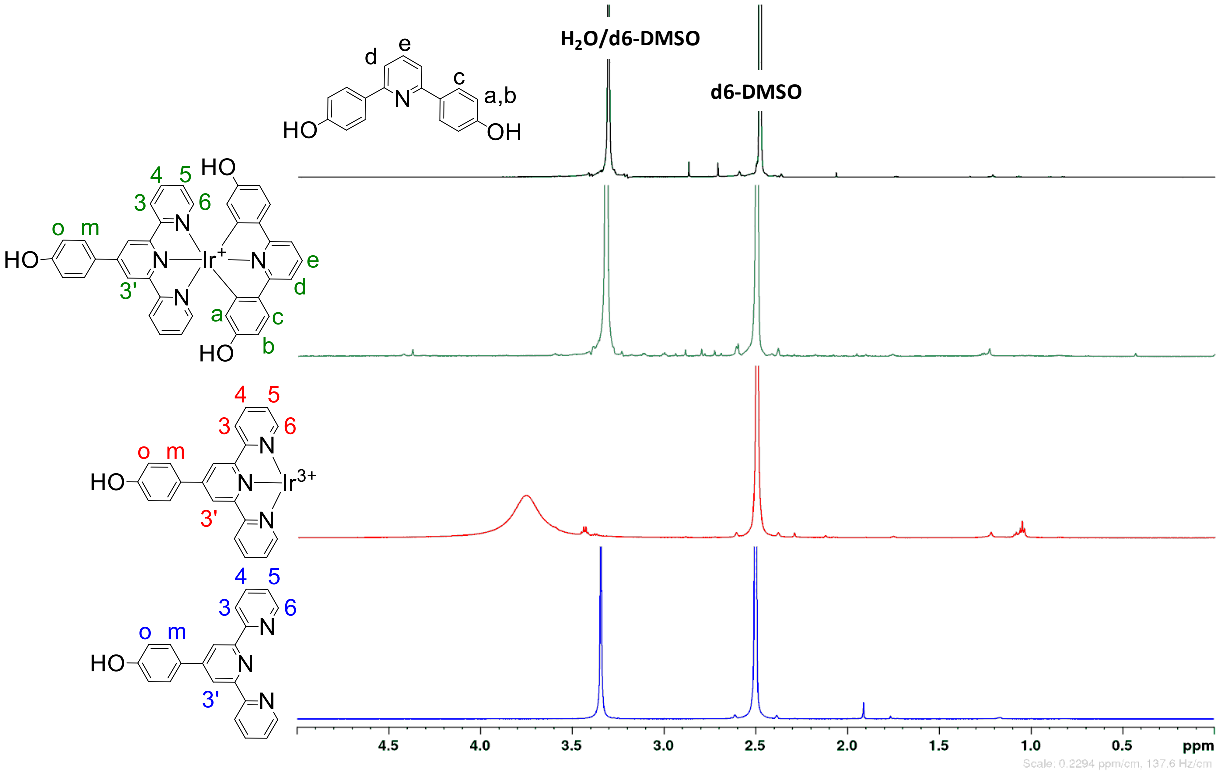

Figure S1. ^{1}H-NMR spectra area 5-0 ppm, of **HOtpy**, **HOtpy-IrCl3**, **HOtpy-Ir-HOpy** and **HOpy** all in DMSO-d6.

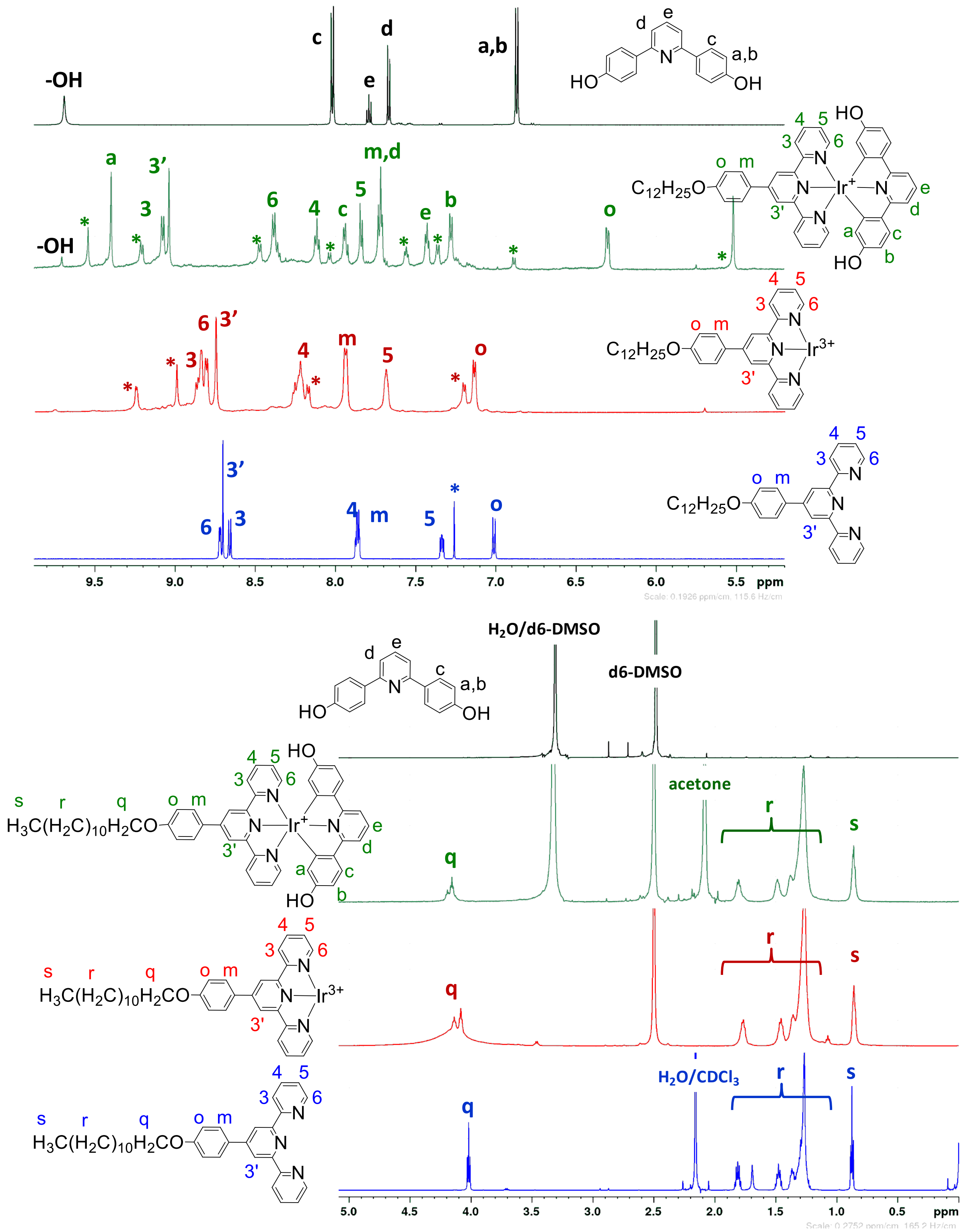

Figure S2. ^{1}H- NMR spectra of **C12Otpy** in $CDCl_3$, **C12Otpy-IrCl3** in DMSO-*d6* and **C12Otpy-Ir-HOpy** in DMSO-*d6*. Peaks denoted with asterisks refer to alternative complexation modes or residual solvent traces.

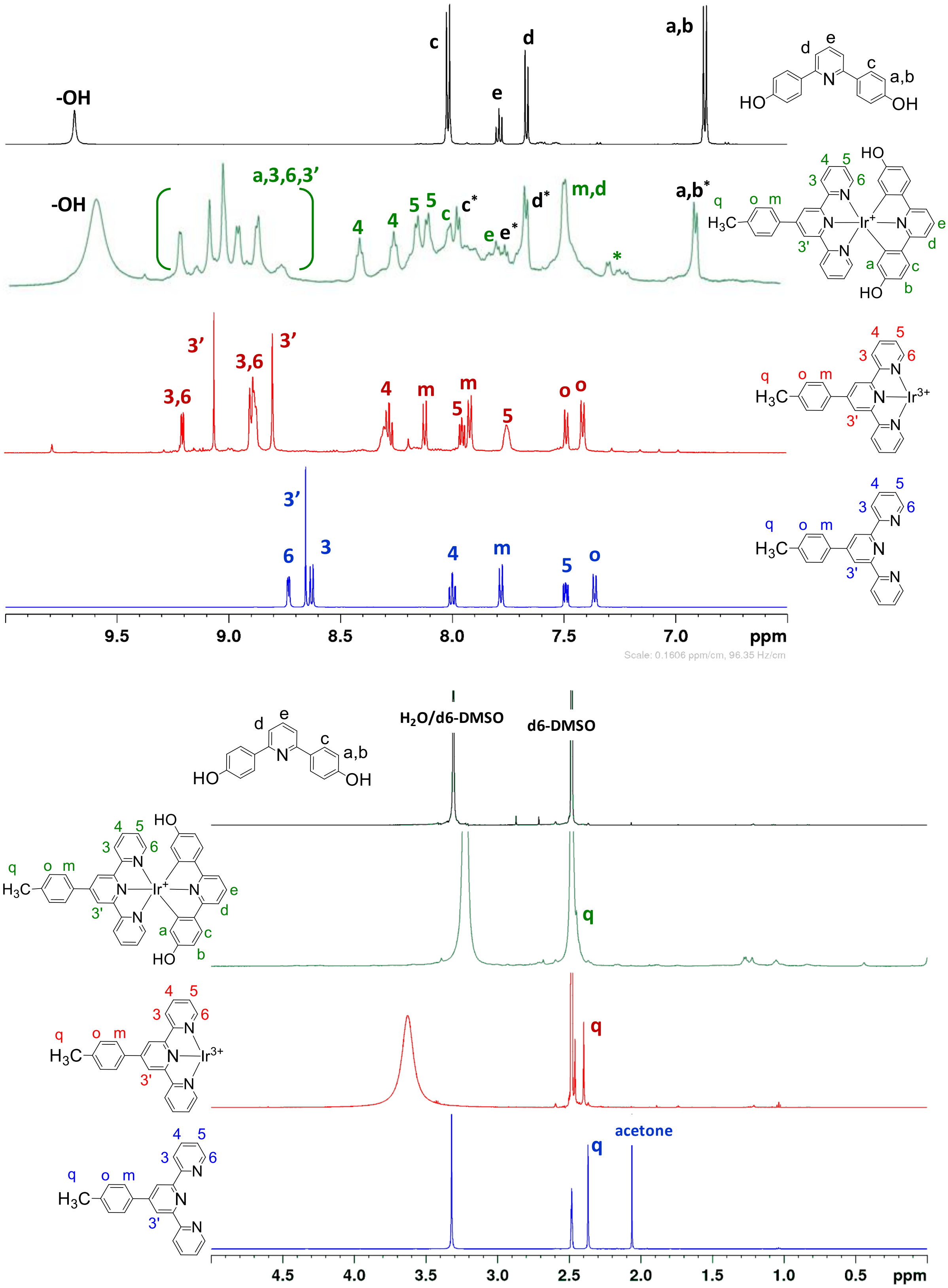

Figure S3. ^{1}H- NMR spectra of **CH₃tpy** in DMSO-*d6*, **CH₃tpy-IrCl₃** in DMSO-*d6* and **CH₃tpy-Ir-HOpy** in DMSO-*d₆*.

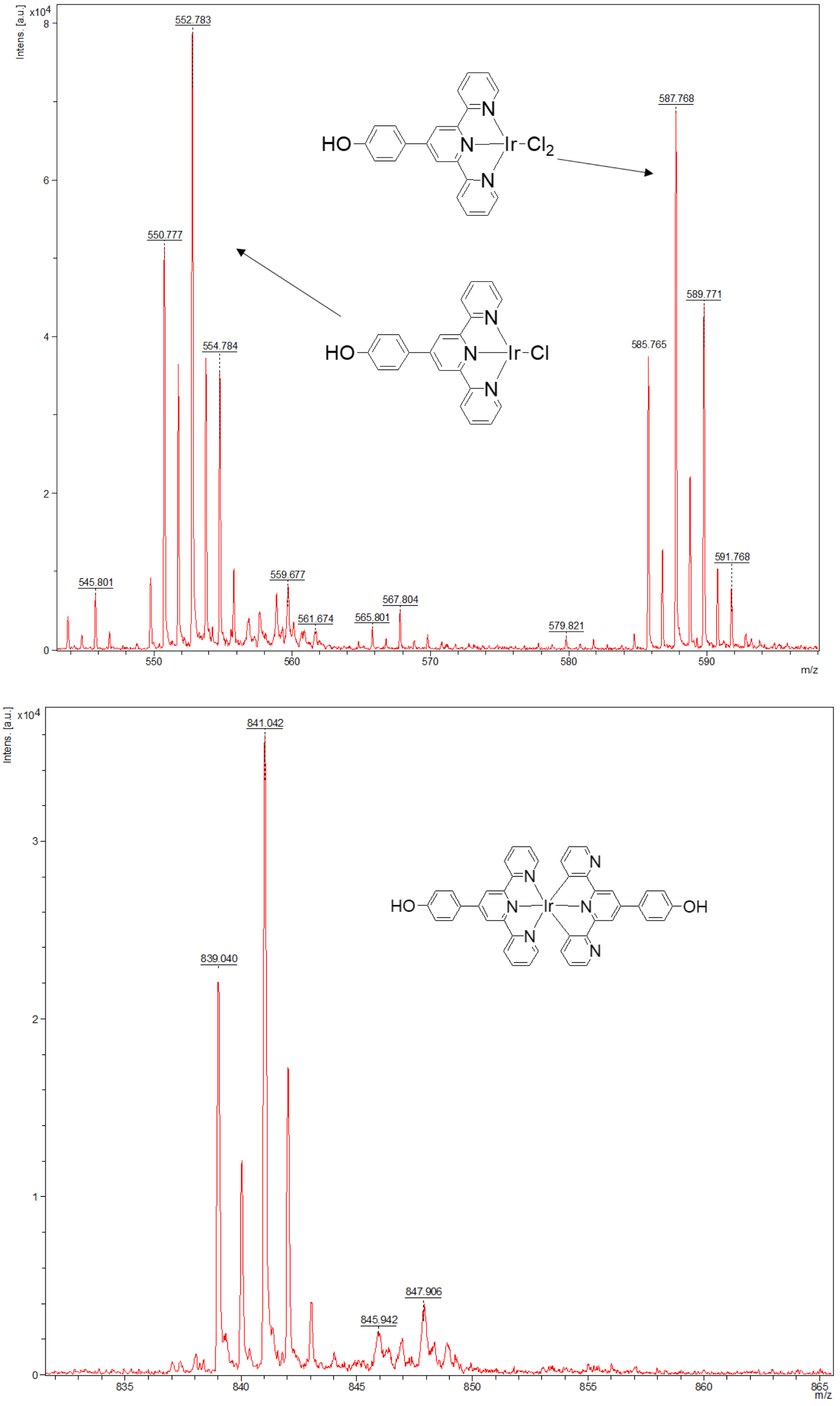

Figure S4. LDI – TOF/MS spectra of **HOtpy-IrCl$_3$.**

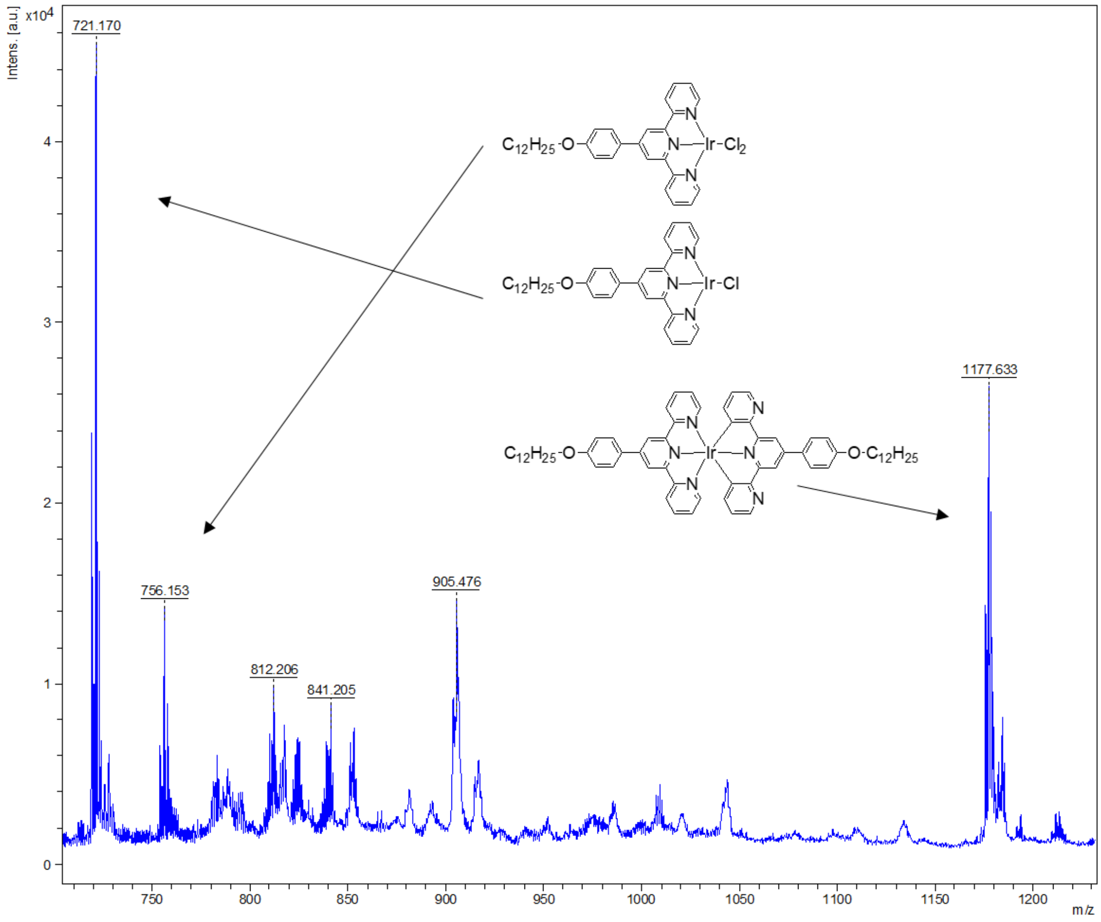

Figure S5. LDI – TOF/MS spectra of **C_{12}Otpy-IrCl_3**

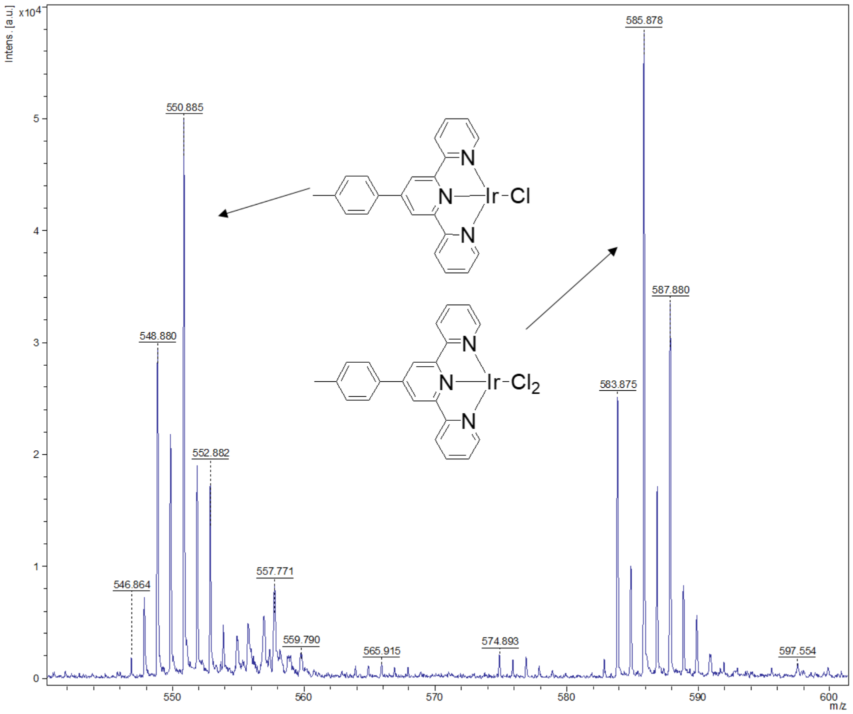

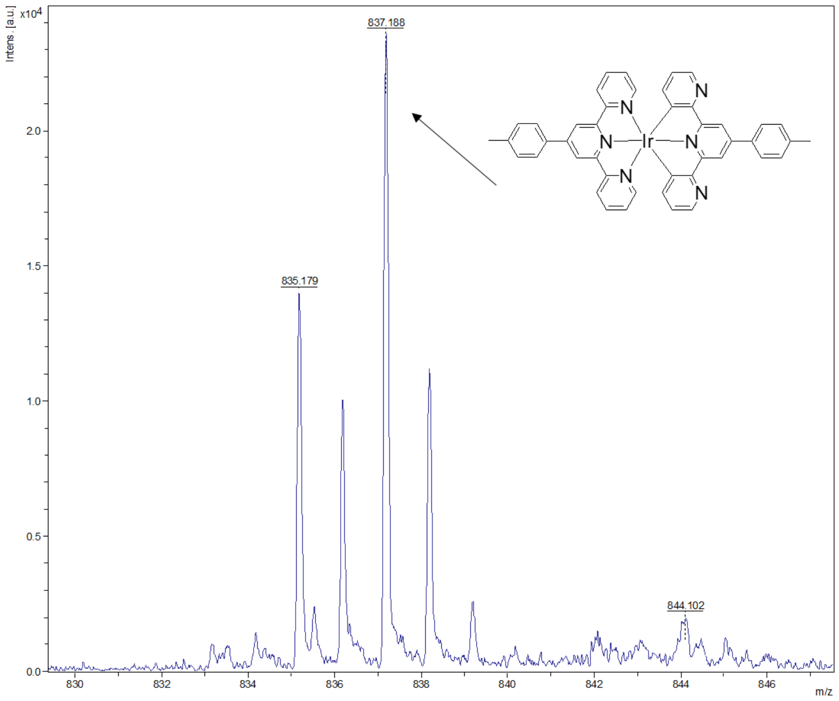

Figure S6. LDI – TOF/MS spectra of **CH_3tpy-IrCl_3**

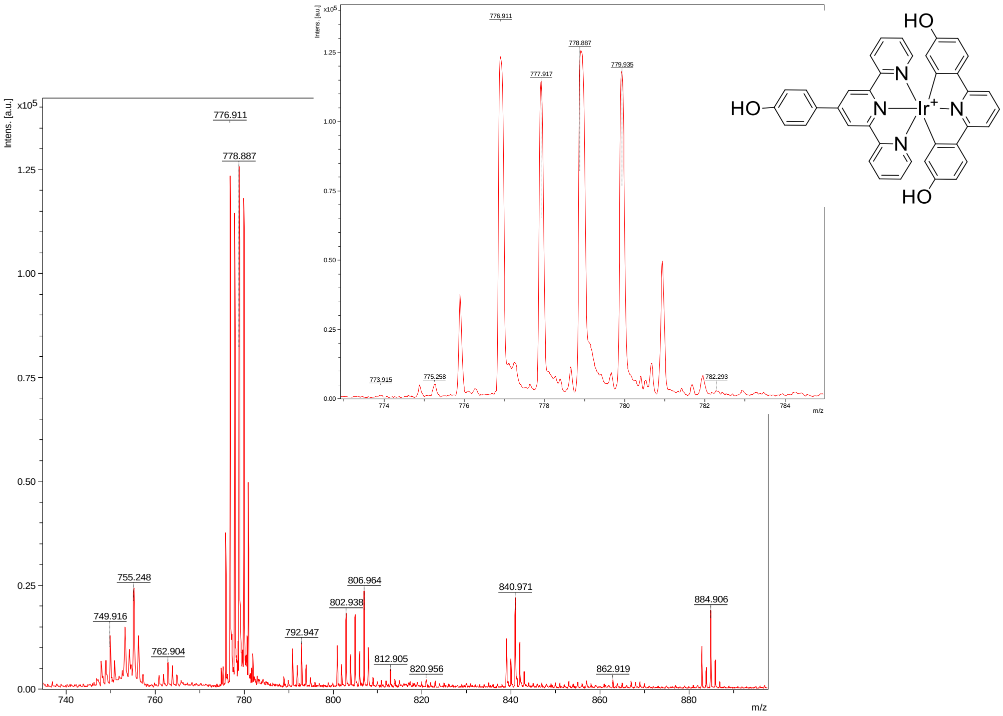

Figure S7. LDI – TOF/MS spectra of **HOtpy-Ir-HOpy**

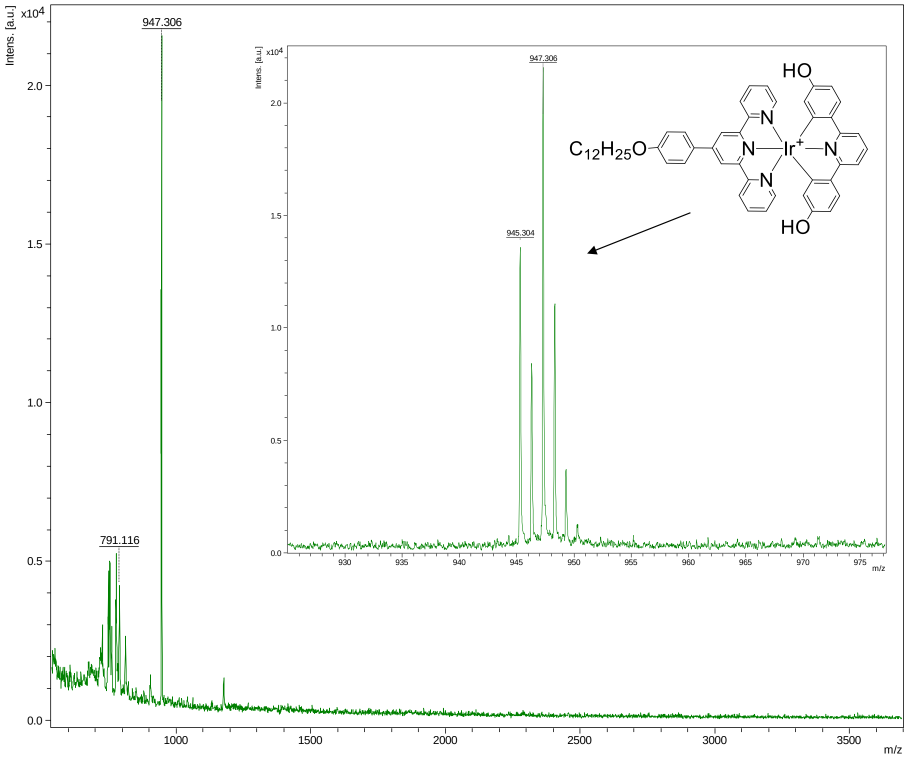

Figure S8. LDI-TOF MS spectra of **C_{12}Otpy-Ir-HOpy.**

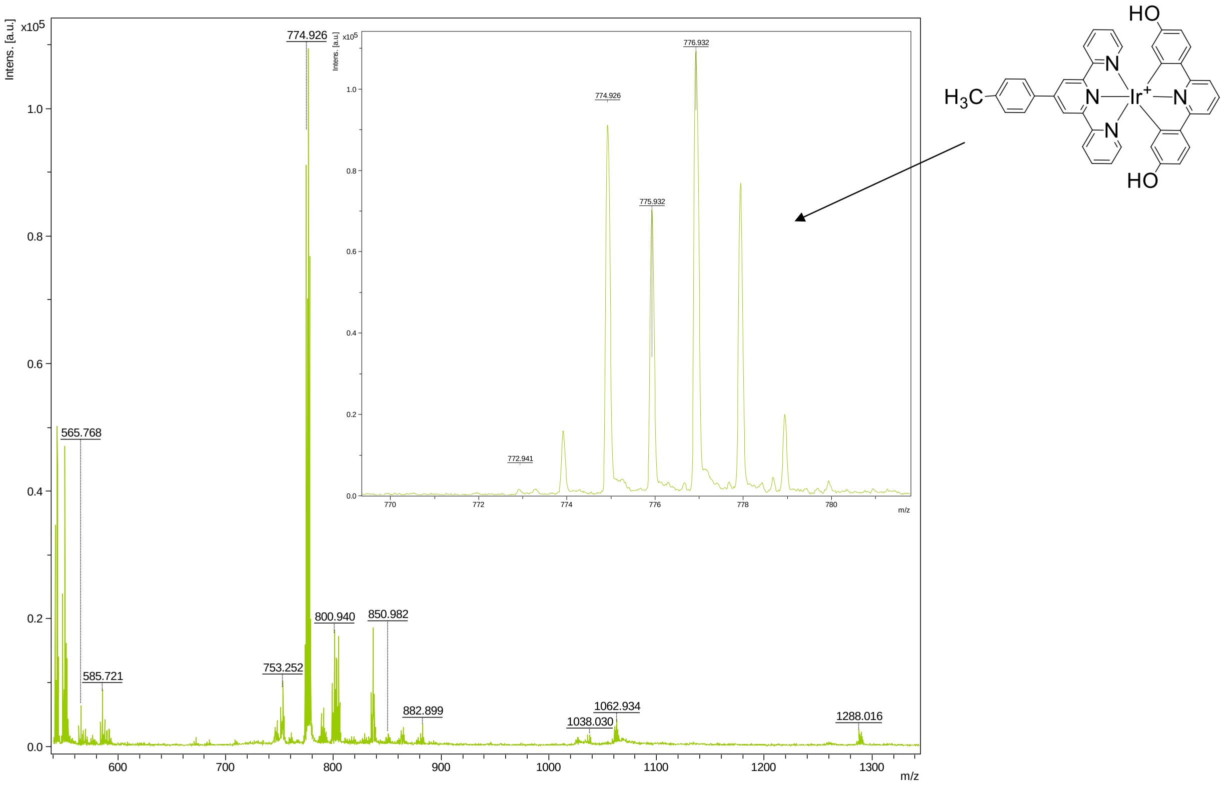

Figure S9. LDI – TOF/MS spectra of **CH₃tpy-Ir-HOpy**

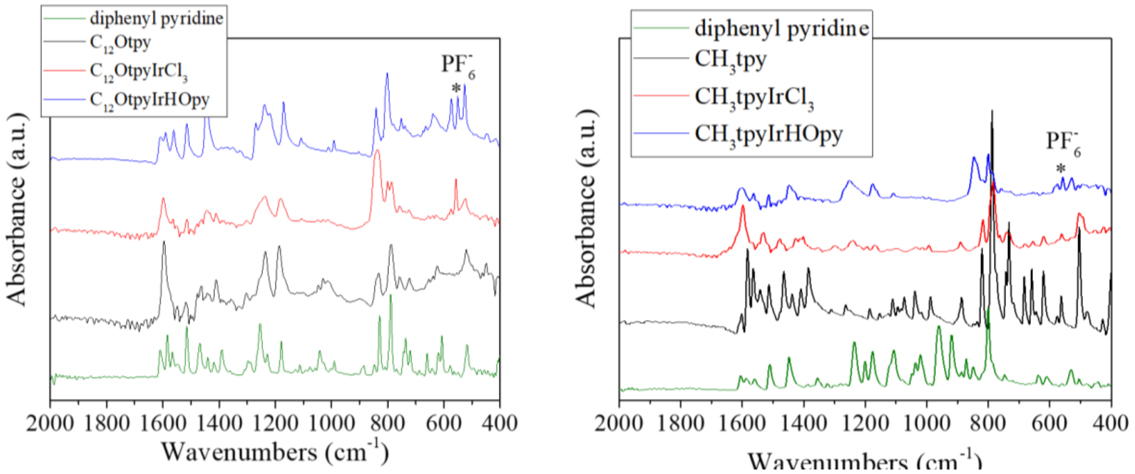

Figure S10. ATR spectra of the **monomeric Ir(III) complexes** based on the **C₁₂Otpy** and **CH₃tpy** ligands.

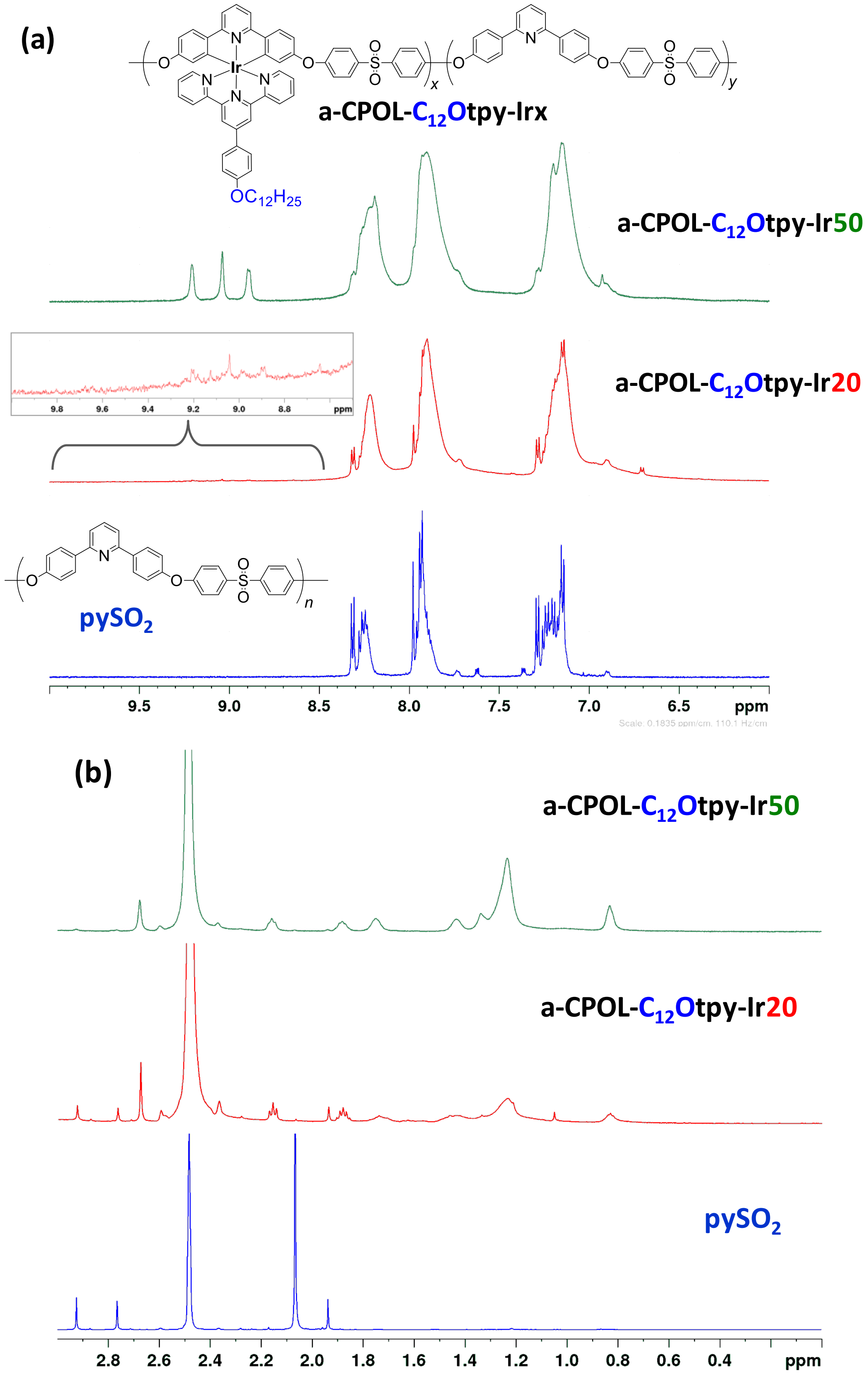

Figure S11. ^{1}H-NMR spectra of **a-CPOL-C$_{12}$Otpy-Irx** (where x=20% red and x=50% green) prepared by the "post polymerization" complexation method, and of the uncomplexated homopolymer ligand **pySO$_2$** in DMSO-d_6. **(a)** shows the region of 10-6 ppm and **(b)** the region 3-0 ppm. The inset in **(a)** shows a magnification of the 10ppm – 8.5ppm area of the **a-CPOL-C$_{12}$Otpy-Ir20** spectrum.

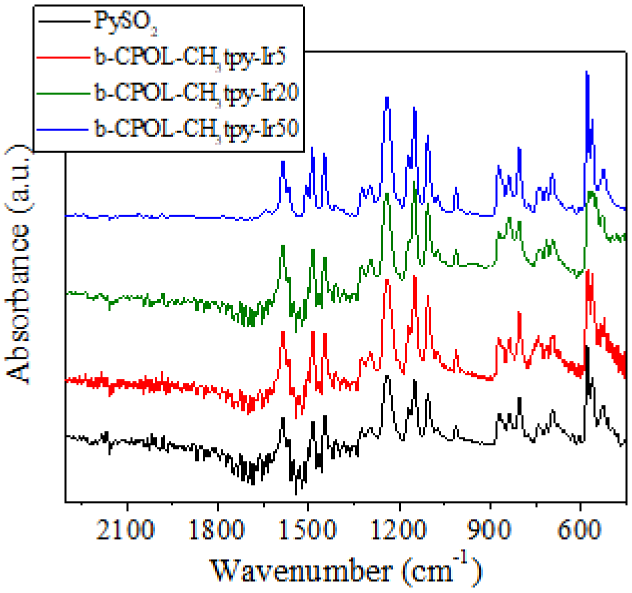

Figure S12. ATR spectra of the polymeric Ir(III) complexes **b-CPOL-CH$_3$tpy-Irx.**

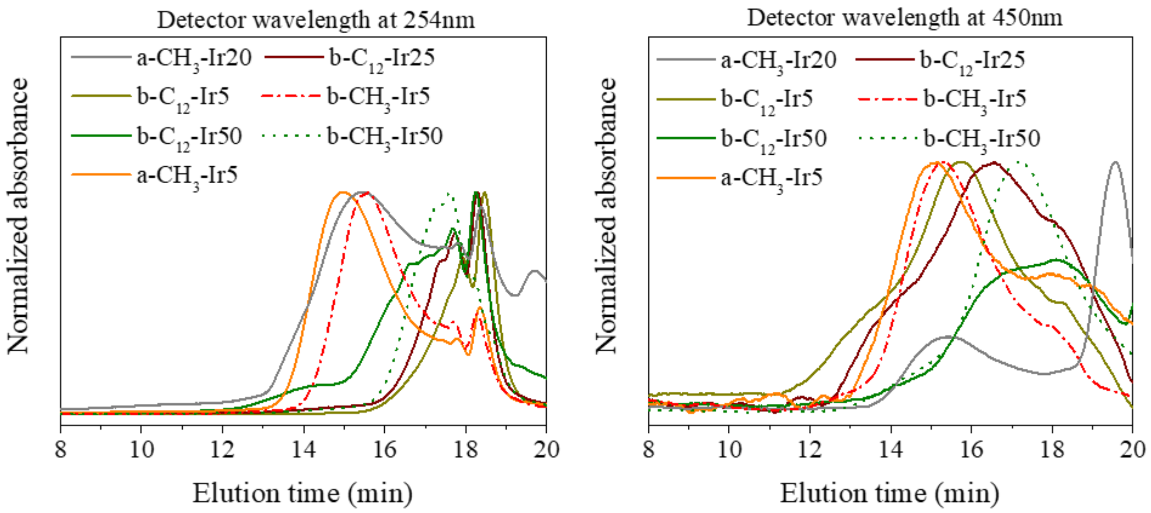

Figure S13. GPC trace of **a-CPOL-Rtpy-Irx** (indicated as **a-R-Irx**) and **b-CPOL-Rtpy-Irx** (indicated as **b-R-Irx**) with the detector set at 254nm (left) and at 450nm (right).

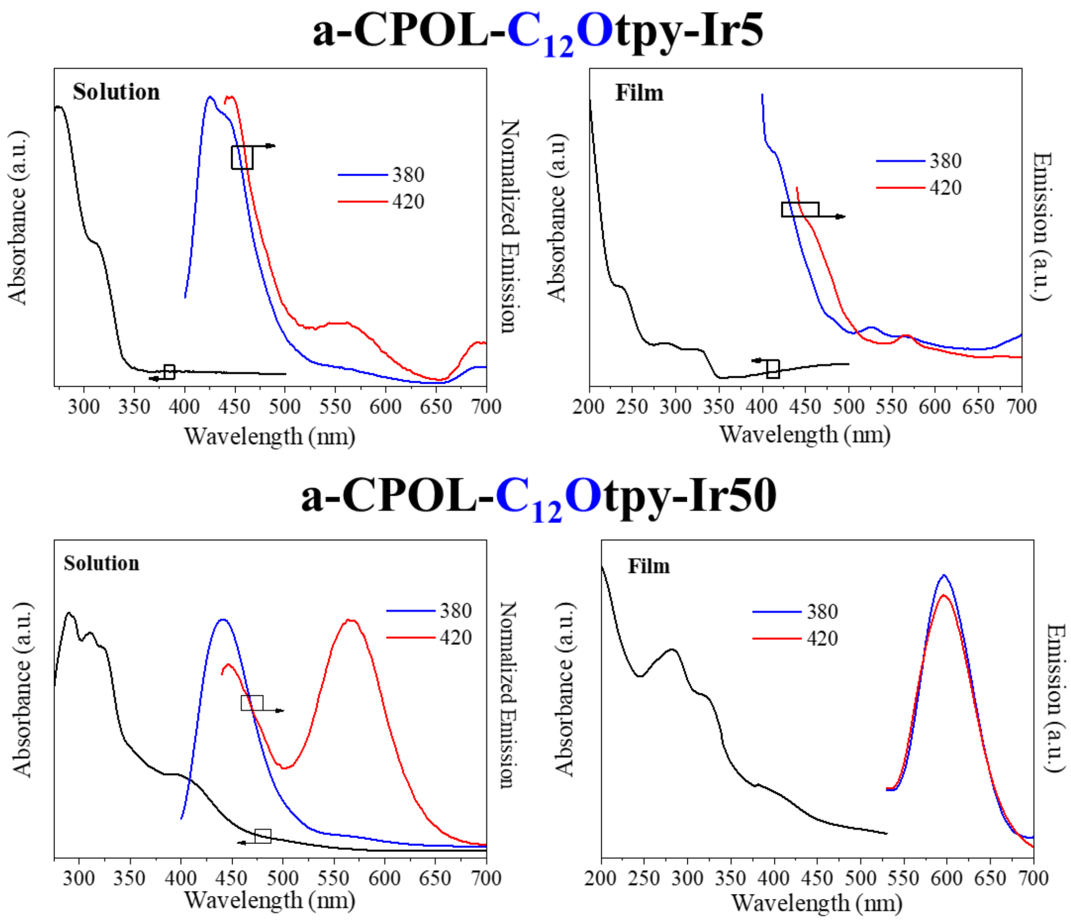

Figure S14. Absorption (black) and emission (color) spectra of the polymeric complexes **a-CPOL-C12Otpy-Irx** where **x=5, 50** recorded in 1,1′,2,2′-TCE solution (left) and thin film (right)

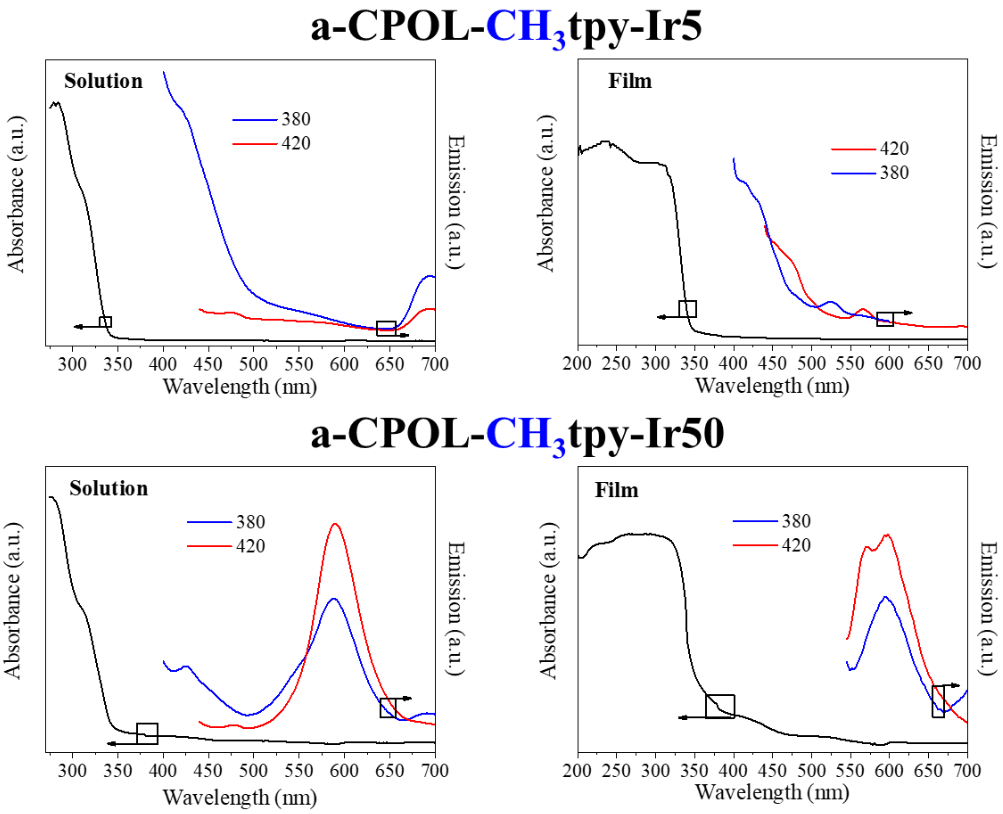

Figure S15. Absorption (black) and emission (colored) spectra of the polymeric complexes **a-CPOL-CH3tpy-Irx** where **x=5, 50** recorded in 1,1′,2,2′-TCE solution (left) and thin film (right)

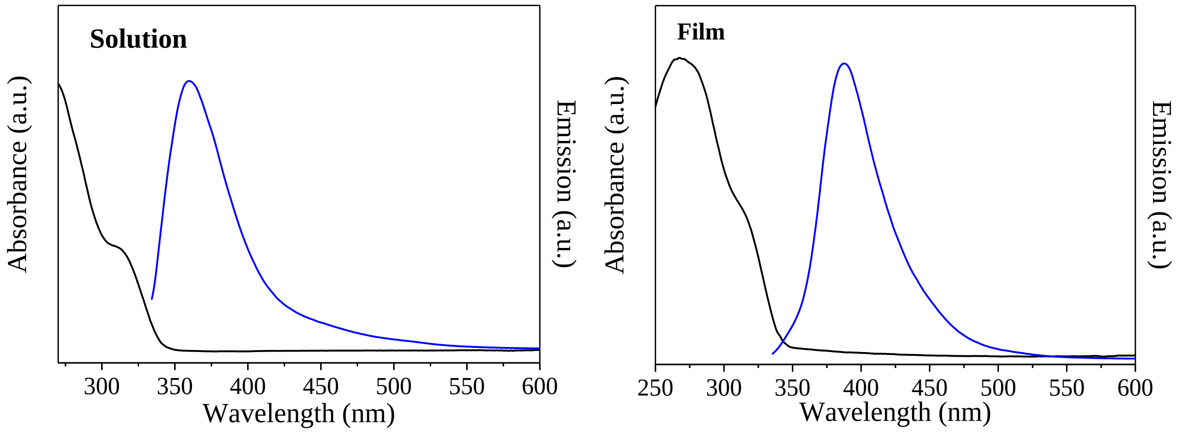

Figure S16. Absorption and emission spectra of **pySO2** in (a) solution using DMA and in (b) thin film form

© 2020 by the authors. Submitted for possible open access publication under the terms and conditions of the Creative Commons Attribution (CC BY) license (http://creativecommons.org/licenses/by/4.0/).
